# Research on sand body architecture at the intersection of a bidirectional sedimentary system in the Jiyuan area of Ordos Basin

**DOI:** 10.1038/s41598-023-28302-y

**Published:** 2023-01-21

**Authors:** Qiang Tong, Dongbo He, Zhaohui Xia, Jixin Huang, Yunbo Li, Fang Xu, Songwei Guo

**Affiliations:** Research Institute of Petroleum Exploration and Development, PetroChina, Beijing, 100083 China

**Keywords:** Fossil fuels, Petrol

## Abstract

The exploration and development of the dual-provenance lower assemblage of the Yanchang Formation in the Jiyuan area has progressed rapidly. At the intersection of this bidirectional provenance system, a complex and variable spatial combination of sand bodies formed, resulting in significant structural heterogeneity in the development and distribution of reservoirs. Based on previous studies, this paper combines core data and logging data with a large number of analytical tests and production performance data to carry out research on the Chang 8_2_–Chang 9 reservoir group in the lower assemblage of the Yanchang Formation in the Shijiawan-Buziwan area. Based on the analysis of sedimentary conditions, the sand body development pattern at the intersection of the bidirectional sedimentary system in the study area was analysed by stepwise dissection of the sand body architecture. After the types and characteristics of the 4th- to 5th-level architectural elements were determined, the spatial distribution of the combinations of these elements was assessed and combined with logging discriminant analysis and geometric shape prediction methods to identify a ‘prism’ architectural distribution pattern. The architectural elements are connected with the distribution of diagenetic facies, the spatial distribution patterns of different types of diagenetic facies under the constraints of the architecture are summarized by region, and the locations of potential favourable reservoir development are discussed. The results show that the degree of superposition and combination of the eight skeletal architectural elements in the target layers gradually deteriorate from the bottom to the top. In addition, the development scale and degree of architectural elements in the braided river delta system in the west are better than those in the meandering river delta system in the east. In the different sedimentary areas, the spatial combination styles of the architectural elements are quite different, and the combination of these elements gradually changes from a combination of braided channels (FA1) and abandoned channels (FA2) to a combination of underwater distributary channels (FA4). Matching of the distribution of diagenetic facies with the distribution of architectural elements reveals that the diagenetic facies dominated by intergranular pores and dissolution pores (associated with good reservoir physical properties) are usually found at the bottom or in the lower to middle parts of the skeletal architectural elements.

## Introduction

With the continuous breakthroughs in the theory and practice of tight sandstone oil and gas exploration and development, the lower assemblage of the Yanchang Formation in the Ordos Basin has attracted much attention^1–3^, especially in terms of source and reservoir characteristics and sedimentary evolution^4–7^, diagenesis and reservoir-forming relationships, and reservoir control mechanisms^8–10^. For example, in the representative Jiyuan Oilfield, to find a strategic succession, the composition of the oil-bearing Chang 8_2_–Chang 9 formation in the lower part has been the focus. However, at present, a series of problems, such as the relatively slow progress in exploration and development and the poor reservoir evaluation effects, have been encountered.

**Figure 1:**
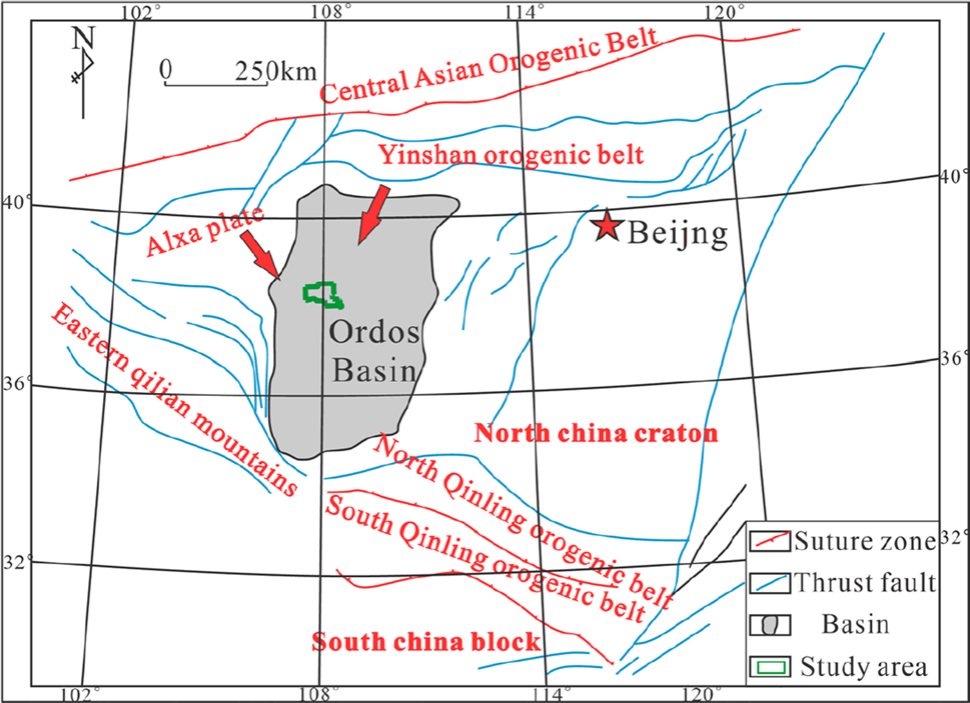
Distribution of the bidirectional provenance system in the Jiyuan area of the Ordos Basin, modified from reference^13^. Created using CorelDRAW-X9 21.2.0.706 (http://www.coreldraw.com/cn/).

First, the provenance direction of the Jiyuan area is mainly controlled by the provenance system to the northwest^9,11^, but the sediments in the eastern and western areas and the associated reservoir characteristics are significantly different^12^, which is speculated to be the result of the influence of the provenance system to the northeast to some extent (Fig. 1)^13,14^.

Previous studies on the bidirectional provenance system are relatively scarce, and relevant research methods are relatively rare, among which sink experiments are representative^15,16^. Most previous studies are based on well, core, and physical property data, using heavy mineral analysis, thin section, and X-ray diffraction analysis methods to distinguish provenance directions to characterize sedimentary microfacies and sand body types^17–19^. A few studies have used seismic data and geochemistry to analyse the sedimentary sequences and accumulation conditions and how they relate to the sediment supply patterns^20–22^. Second, although the differences in sedimentary genesis, sand body types, and structural characteristics have attracted attention^1,11^, most studies have focused on the sub-oil-bearing Chang 8_1_ unit^23,24^, which has led to a lack of in-depth research on underlying layers. Finally, the evolution of the sedimentary system and the architectural combination of sand bodies formed by the intersection of this type of bidirectional provenance system have not been analysed, resulting in poor understanding of the reservoir development and oil distribution under its control.

In response to the above problems, this paper uses abundant core data, well logging data, dynamic data, and statistical software to study the sedimentary characteristics and sand body architecture of the oil-bearing Chang 8_2_–Chang 9 formation in the Shijiawan-Buziwan area of the Jiyuan Oilfield. Then, the sedimentary architecture model at the intersection of the bidirectional provenance system is established, and the physical properties and diagenetic facies of the reservoir are analysed based on core physical property analysis, thin sections and scanning electron microscopy. In addition, the spatial constraint relationship between various architectural element combinations and different diagenetic facies is clarified. Last but not least, based on this work, the pattern of oil enrichment from the perspective of sand body architecture is discussed, thereby providing a certain theoretical basis for exploration and development in this type of sedimentary background.

## Regional geological overview

The Ordos Basin in the western part of the North China platform is a stable multicycle craton basin. From the early Palaeozoic to the Mesozoic, the basin experienced the following three evolutionary stages: (1) Cambrian to Early Ordovician, when different discrete continental margins developed; (2) Middle Ordovician to Middle Triassic, when a convergent continental margin developed; and (3) Late Triassic to Early Cretaceous, when the remnants of the cratonic basin developed^25^. The tectonic belt on the western margin of the basin is located between the Alxa massif and the Ordos basin, and its southwestern boundary corresponds to the northeastern boundary of the Qinghai–Tibet Plateau. The tectonic evolution was directly affected by the development of the Qinghai–Tibet Plateau. In particular, the structural characteristics of the basin, especially the western edge, can be generally characterized by east‒west zonation and north–south segmentation^26^. The northern part of the basin is the Xingmeng orogenic belt, and the Yinshan Yanshan orogenic belt crosses the northern margin of the North China Craton. During the Indosinian-Yanshanian period, the crust in this area experienced multiple phases of compressional and extensional deformation, forming a thrust nappe tectonic system. As it is located at the convergence of three continental blocks, namely, North China, South China and the northeastern margin of the Qinghai–Tibet Plateau, its western margin is strongly deformed due to the eastward flow of deep materials from the northeastern margin of the Qinghai–Tibet Plateau^27,28^.

**Figure 2:**
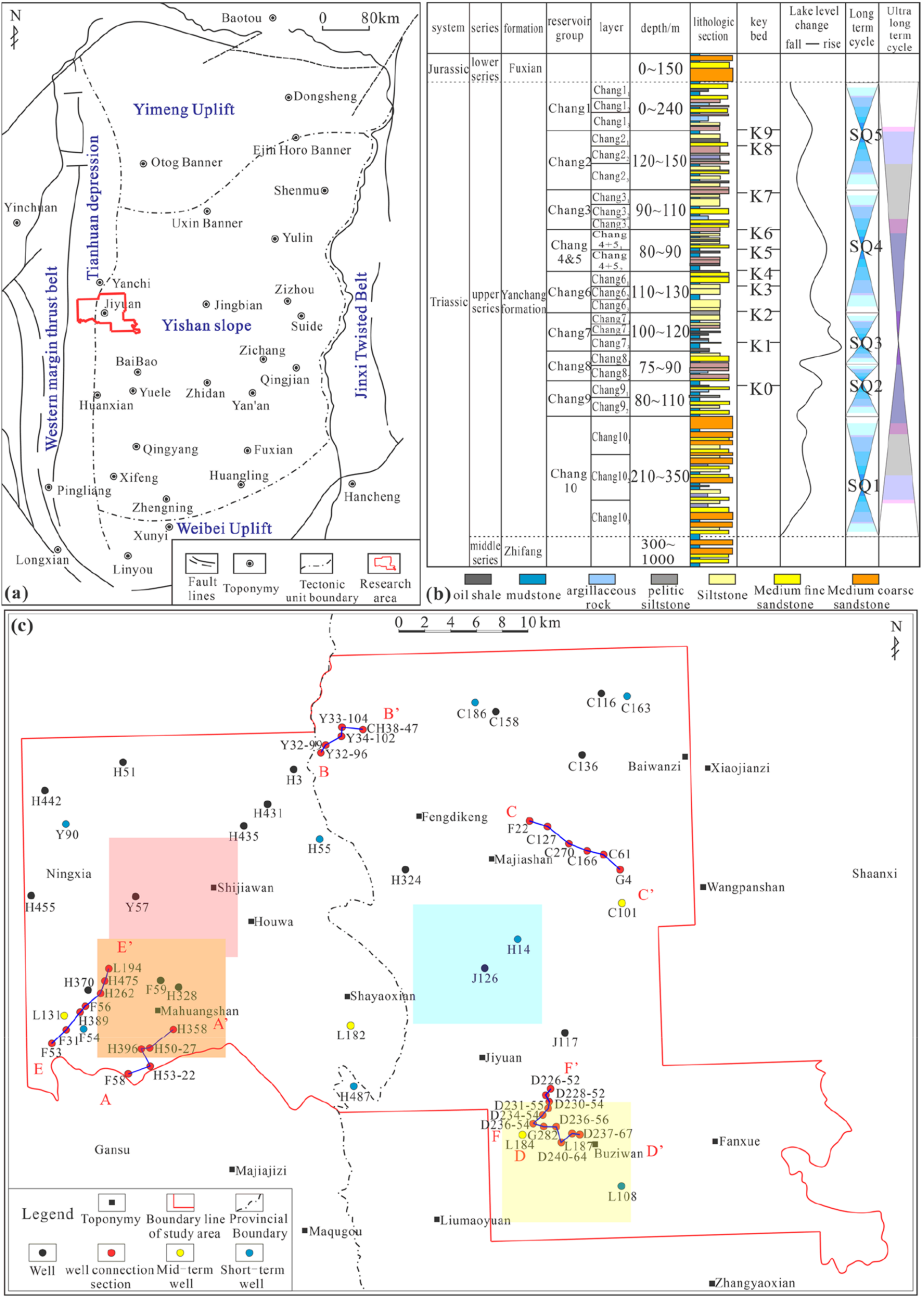
(**a**) Basin structural division and study area location (modified from reference^13^. Created using CorelDRAW-X9 21.2.0.706 (http://www.coreldraw.com/cn/); (**b**) Yanchang Formation sequence stratigraphic division (modified from reference^3^. Created using CorelDRAW-X9 21.2.0.706 (http://www.coreldraw.com/cn/) (**c**) Well location distribution in the study area (AA' is the well connection profile of F58-H358; BB’ is the well connection profile of Y32-99-CH38-47; CC’ is the well connection profile of F22-G4; DD’ is the well connection profile of D236-54-D237-67; EE’ is the well connection profile of F53-L194; FF’ is the well connection profile of D236-54-D226-52. The red, blue, orange and yellow shaded areas correspond to the extents of the skeleton architectural elements FA1, FA6, FA2 and FA4 in "The spatial distribution characteristics of architectural elements" Section, respectively.) Created using GeoMap v3.6 (http://www.jurassic.com.cn/).

The Ordos Basin was an inland lake basin in the Mesozoic. The Jiyuan area is located in the central and western parts of the basin^29^, and its structure spans the Tianhuan Depression and the Yishan Slope (Fig. 2a)^13^. Affected by the Indo-China, Yanshan, and Himalayan orogenic events, the current main body is a gentle west-dipping monocline^30^, forming a microstructural feature with many nearly east‒west nose-like uplifts^31^. The Yanchang Formation is divided into 10 oil-bearing formations from bottom to top^32^. During the deposition of the oil-bearing Chang 9 formation to the sub-oil-bearing Chang 8_2_ formation, the lake basin experienced stable subsidence within a background of water invasion and greatly expanded over time^24^. As a whole, these units belong to the third-order sequence SQ2 (Fig. 2b)^3^, with the oil-bearing Chang 9 formation corresponding to a low-stand systems tract and the sub-oil-bearing Chang 8_2_ formation corresponding to a high-stand systems tract^33^. The study area extends from the Wangpanshan-Fanxue area in the east to the Mahuangshan area in the west and from the Buziwan area in the south to the Xiliang-Youfangzhuang area in the north. The study area covers an area of approximately 1857 km^2^ and includes more than 750 wells. The locations of these wells are shown in Fig. 2c.

The Upper Triassic Yanchang Formation developed as a set of terrigenous clastic deposits dominated by deltaic lacustrine facies^34^. In the context of the expansion of the lake basin, the study area simultaneously received sediment from the Alxa ancient land to the northwest and the Yinshan ancient land to the northeast^35,36^. Therefore, the oil-bearing Chang 9 formation in the study area developed in both braided river delta and meandering river delta depositional environments, while the sub-oil-bearing Chang 8_2_ formation developed in shallow-water delta depositional environments^9^. However, according to analysis and comparison by the authors of this paper, there are still unanswered questions about the sedimentary environment of the study area. On the one hand, some scholars believe that the sub-oil-bearing Chang 8_2_ formation in the Jiyuan area formed in a beach bar sedimentary environment^37^, not a shallow delta front environment. Furthermore, the boundary between braided river delta and meandering river delta deposits in the oil-bearing Chang 9 formation is not clear, and the types of subfacies and microfacies are not uniform^38,39^.

## Methodology

### Data collection

The data collected in this paper are from the Exploration and Development Research Institute of PetroChina Changqing Oilfield Company. They include well location data, logging data, core photos, physical properties of core analysis, particle size analysis, and thin section data.

### Petrological characteristics

The petrological characteristics analysis is based on core photographs, core analysis physical properties and thin section data, and the results are plotted on a rock structure ternary diagram by using the three terminal element method in petrology. The cumulative distribution curve of particle size comes from the collected graphical particle size analysis report.

### Analysis of the hierarchical structure of architectural elements

The method of hierarchical structure analysis is mainly based on Miall and Wu's research methods and ideas^40–42^. The specific research methods are as follows. Through research on the classification of architectural interfaces, the identification and analysis of lithofacies types, and the characterization of architectural elements, the various levels of sedimentary units after sequence stratigraphic division can be transformed into corresponding sequence architectural elements^41,42^. After consideration of the relationships between various levels of cyclic structures and their corresponding stratigraphic units, it is concluded that the short-term cyclic structures identified above correspond to 5th-level architectural elements, forming a 5th-level sequence. The sequence has a certain degree of isochronism only under local dense well pattern conditions. The ultrashort-term cycles formed by further division correspond to 4th-level architectural elements at the genetic scale, which is called the single sand body level. Monogenetic sand bodies of the same period have the same sedimentary genesis (Table 1).

**Table 1:** Grade division scheme of architectural elements.

Architecture interface level	Corresponding base level cycle	Architectural element scale	Genetic interpretation
Level 0	–	Lamination	Water ripple
Level 1	–	Layer system/Layer group/Micro bottom shape	Single lithofacies
Level 2	–	Lithofacies/medium bottom shape	Single lithofacies superposition
Level 3	–	Internal accretion of monogenetic sand body	Continuous sedimentary sequence controlled by weak sedimentary discontinuity
Level 4	Ultrashort-term base level cycle	Single microfacies/single origin sand body/large bottom shape	Single sand body such as a single distributary channel or single estuary dam
Level 5	Short-term base level cycle	Single microfacies complex/single channel belt	Compound distributary channels, underwater distributary channels, and estuary dam complexes, etc
Level 6	Medium-term base level cycle	Single-layer microfacies complex/composite channel belt	Overlay of compound distributary channel sand bodies

### Facies architecture interface recognition

The first step of reservoir architecture hierarchy analysis is architectural interface analysis. On the basis of Miall^41,42^, predecessors proposed an analysis method for fluvial facies reservoir architecture, and the identification of architectural interfaces has also been widely reported^43–45^. First, the architectural interfaces of each level are identified by core observation, and the electrical logging curve parameters of the architectural interface are extracted from the electrical logging curve. Then, the electrical characteristics of the architectural interface are summarized to enhance research in all areas.

Allocyclicity and autocyclicity control the formation, distribution, and evolution of architectural elements^46,47^. According to the method of hierarchical structure analysis and the results of high-resolution sequence stratigraphy, it is necessary to further refine the short-term cycles to ultrashort-term cycles. The corresponding 3rd–5th-level architectural elements belong to the facies architecture^48^. Therefore, it is necessary to establish facies architectural interfaces that can finely characterize the sedimentary discontinuities based on calibration of the core and logging curves in order to establish the electric feature identification standard for the studied rocks^36^.

### Method of predicting the geometry of the river delta system

**Figure 3:**
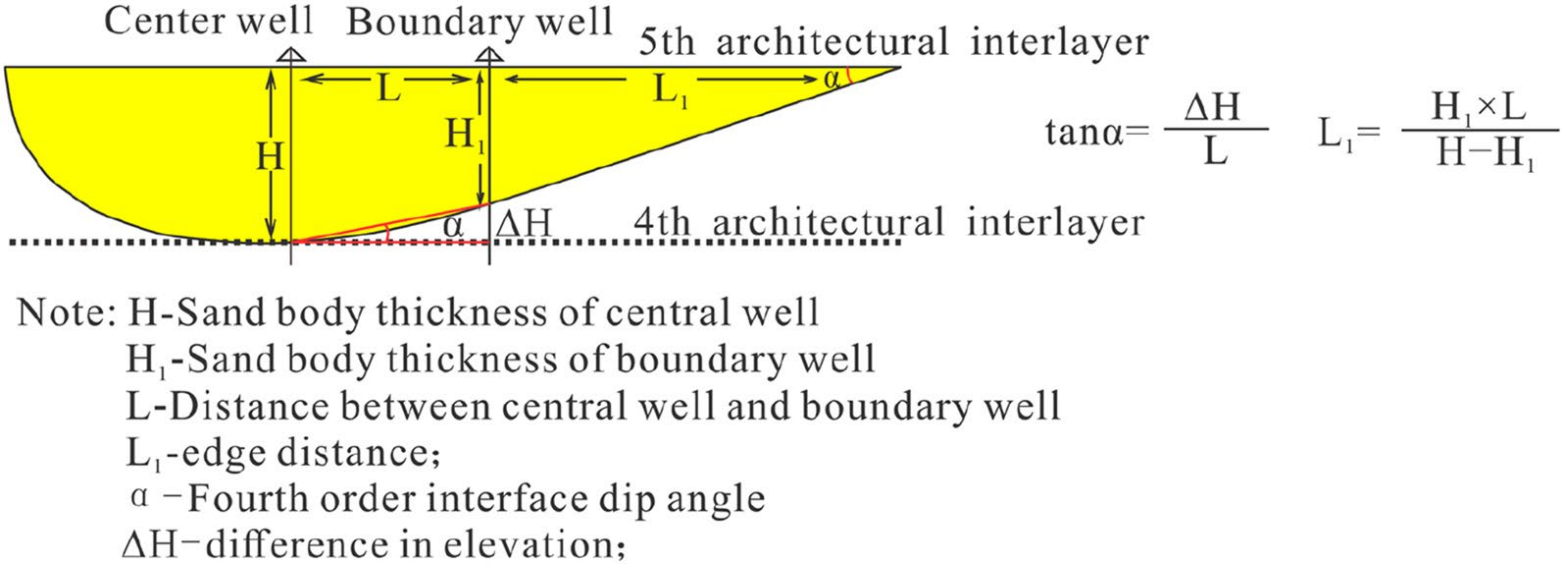
Geometric form prediction method of architectural elements. *Note* This model is a single channel thickness prediction model (modified from reference^49^).

Considering that the Yan River section and Rui River section are far from the study area and that there is obvious uncertainty in the characterization method of modern sediments due to the differences in sedimentary bodies, this paper selects the dense well pattern method in combination with a prediction model to build a geological knowledge base of sand body architectural parameters^49,50^ in order to carry out geometric morphology characterization (Fig. 3). In addition, the connectivity is verified with the help of production dynamic data, and the correction of the geometric shape predictions is realized^51^.

### Method of quantitative logging identification of sand body architectural elements based on genetic analysis

Limited by the number of cored wells, it is necessary to establish a quantitative identification method of sand body architectural elements based on genetic analysis to accurately and quickly interpolate the data to noncored wells. The manual sorting and extracting of the logging data from many cored well architectural elements reveals that the logging curve parameters of various elements show a certain regularity. The reason is that there are significant differences in the lithology and lithofacies combinations of each type of architectural element, which result in differences in the logging curve parameters.

### Fisher discriminant analysis methodology

The Fisher discriminant method uses statistical concepts and methods to extract electrical measurement data from different architectural elements and then uses SPSS (Statistical Product Service Solutions) software for linear regression analysis to obtain a discriminant function. Discriminant analysis is then conducted on architectural elements in other wells. After collinearity assessment, the variance inflation factor (VIF) between the parameters should be less than 10, indicating that there is no typical multicollinearity. In such cases, the discriminant analysis is effective.

### Diagenetic facies analysis method

In this paper, the analysis of diagenetic facies is mainly based on the analysis method of dominant facies. Specifically, the main type of diagenesis of a sample is extracted by observing and analysing thin sections. Through the analysis of typical thin section data from cored wells, the dominant diagenetic facies types are summarized. After core positioning, the vertical distribution position of the diagenetic facies identified in the thin sections from the architectural elements should be calibrated. After sorting, the distribution rules of different diagenetic facies in the architectural elements are obtained. Furthermore, the relationship between architectural elements and the diagenetic facies distribution is established to further analyse the relationship between the diagenetic facies development characteristics and the sedimentary environment within the context of different architectural element combination modes to provide a basis for subsequent reservoir characteristics research. As this paper focuses on the development characteristics of the dominant diagenetic facies under different distribution modes of architectural elements, the diagenetic facies are not described in detail.

## Difference boundaries of sedimentary characteristics under the bidirectional provenance system

The characteristics of the oil reservoirs in the sub-oil-bearing Chang 8_2_ formation to the oil-bearing Chang 9 formation of the Yanchang Formation in the Jiyuan Oilfield are complicated, and the effective factors controlling the relationship between structure and lithology are still relatively poorly constrained. The feedback on oilfield development status suggests that the geological characteristics in the east and west of the study area are quite different, and it is speculated that this difference is the result of the different sedimentary environments resulting from a dual provenance system. There is clear evidence for a bidirectional provenance system, such as light and heavy minerals, rare earth elements, and quartz cathodoluminescence^35^. The existence of a braided river delta depositional environment in the west and a meandering river delta depositional environment in the east has also been discussed^52^. However, a detailed analysis of the boundary between sedimentary characteristics has yet to be performed.

### Petrological characteristics

**Figure 4:**
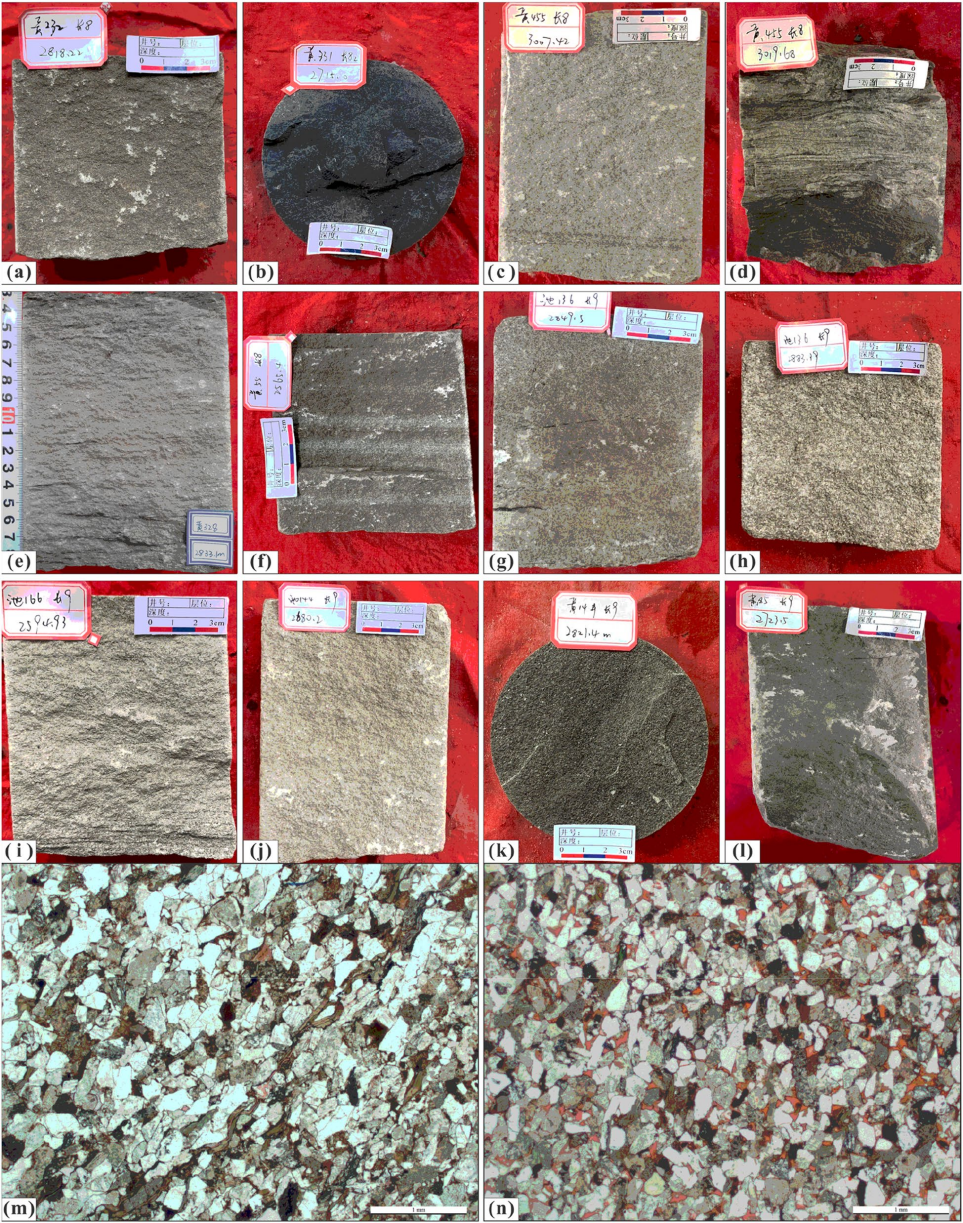
Lithologic and colour characteristics. *Note* (**a**) Grey‒brown oil-bearing fine sandstone, Well Huang-232, Chang 8_2_, 2818.22 m; (**b**) Grey‒black mudstone, Well Huang-331 Chang 8_2_, 2715.0 m; (**c**) Grey‒green fine sandstone, Well Huang-455, Chang 8_2_, 3007.42 m; (**d**) grey‒black argillaceous siltstone, Well Huang-455, Chang 8_2_, 3019.68 m; (**e**) off-white fine sandstone, Well Huang-328, Chang 8_2_, 2833.1 m; (**f**) Greyish brown/grey‒white fine sandstone, Well Huang-55, Chang 8_2_, 2565.4 m; (**g**) Grey‒brown oil-bearing fine sandstone, Well Chi-136, Chang 9, 2849.5 m; (**h**) Grey‒brown oil-bearing medium sandstone, Well Chi-136 Chang 9, 2883.39 m; (**i**) off-white fine sandstone, Well Chi-166 Chang 9, 2594.93 m; (**j**) oil-bearing fine sandstone, Well Chi-144, Chang 9, 2680.2 m; (**k**) Grey‒black mudstone, Well Huang-14, Chang 9, 2821.4 m; (**l**) Grey‒black siltstone, Well Huang-85, Chang 9, 2723.5 m; (**m**) Thin section, Well Huang-55, Chang 8_2_, 2561.6 m; (**n**) Thin section, Well Huang-431, Chang 9, 2700.3 m.

The sandstone is mainly grey and off-white in colour. Grey‒green fine siltstone and siltstone can be seen in the sand–mud transition section, reflecting the partial reducing redox conditions of the sedimentary environment. The portion that developed under weaker hydrodynamic conditions is dominated by dark grey, grey‒black argillaceous siltstone and black silty mudstone, and the colour becomes darker with increasing argillaceous content. The filling degree of the sub-oil-bearing Chang 8_2_ formation is relatively high, and oil spots and even oil leaching, which are greyish brown, can be seen in the core observation results. In contrast, the filling of the oil-bearing Chang 9 formation is weaker, and this unit features mostly oil stains, which are only slightly grey‒brown. In addition, the heterogeneity is significant, with obvious oil content in bands or blocks (Fig. 4a–l). In the image granularity analysis, the sub-oil-bearing Chang 8_2_ formation and the oil-bearing Chang 9 formation are mainly composed of fine sandstone. According to thin sections with the same scaling factor, the grain sizes of the Chang 8_2_ and Chang 9 formations mainly correspond to fine sand, but the comparison between them shows that the local grain size of Chang 8_2_ is smaller than that of Chang 9 to some extent (Fig. 4m, n).

**Figure 5:**
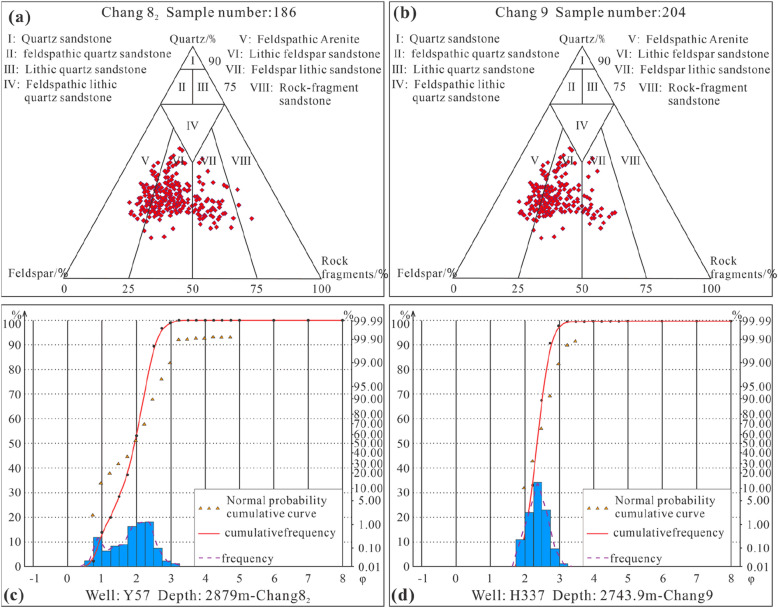
Rock composition and structural characteristics. (**a**) Ternary diagram of rock composition of sub-oil-bearing Chang 8_2_; (**b**) Ternary diagram of rock composition of oil-bearing Chang 9; (**c**) Grain size distribution and cumulative distribution curve of sub-oil-bearing Chang 8_2_ in Well Y57, 2879 m; (**d**) Grain size distribution and cumulative distribution curve of oil-bearing Chang 9 in Well H337, 2743.9 m.

The rock types are dominated by lithic feldspar sandstone and feldspar lithic sandstone. The overall quartz composition is not high, and the compositional maturity is medium (Fig. 5a, b). The cumulative curve of sandstone grain size probability generally presents two-stage characteristics, with significant saltating components, accounting for approximately 70–80%, and less suspended components, accounting for approximately 20–30%, showing a notable inflection point. The clast sorting is relatively poor, and the sedimentary characteristics of traction currents are relatively obvious, reflecting a river delta sedimentary environment.

The image granularity analysis data of the Chang 8_2_ and Chang 9 sediments were input into the Sahu environment discriminant function to obtain the following results^53^. The calculation results of the Chang 8_2_ formation (Y = 18.2794) and Chang 9 formation (Y = 17.6064) conform to a river delta sedimentary environment (Y > 9.8433). Furthermore, based on previous research results and the core observations in this research, it is believed that the Chang 8_2_ formation is more in line with a shallow-water delta sedimentary environment^24^. It can be seen from the cumulative distribution curve of the particle size of the two samples that there is a certain difference in the slope of the fitting line segment representing saltation transport. The slope of the sample from Chang 8_2_ is relatively low, representing relatively poor sorting (Fig. 5c, d).

### Distribution characteristics of sedimentary structures

The oil-bearing Chang 9 formation and sub-oil-bearing Chang 8_2_ formation have various types of sedimentary structures, with significant sedimentary environment indicators, and large-scale cross-bedding is relatively developed, reflecting the strong transport capacity of fluvial water bodies. Additionally, sedimentary structures associated with relatively low-energy partially static water conditions are also common, reflecting the sedimentary environment between river channels or overflow banks. Animal and plant detrital fossils are more common and have obvious carbonization, showing the characteristics of oxidation, suggesting that the violent fluctuation of lake waves caused the front sediments to be often exposed to the water surface. Therefore, the lake shoreline likely experienced frequent migration.

**Figure 6:**
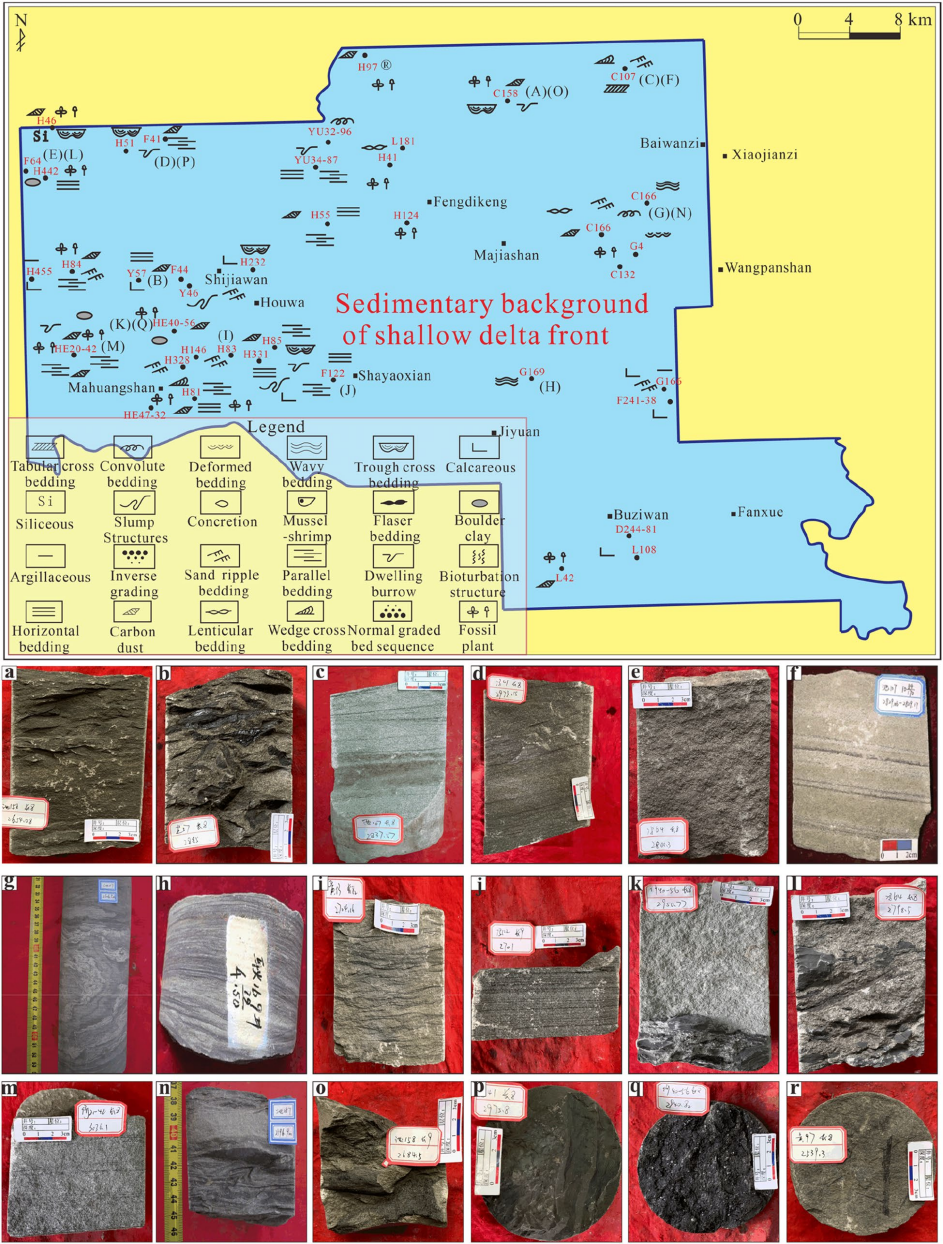
The planar distribution of sedimentary structures in the sub-oil-bearing Chang 8_2_ formation.

The core-based spatial analysis of the sedimentary structures in the sub-oil-bearing Chang 8_2_ formation shows that the wave-formed sand-laminated bedding is widespread, reflecting the wide fluctuation of lake waves (Fig. 6).

A large number of carbonized plant stem debris fossils can be seen in mudstone, and biological burrow structures, such as vertical wormholes, are occasionally seen, reflecting an occasional weakly oxidizing sedimentary environment exposed to water. The wrap-around bedding reflects the slight turbulence at the bottom of the local sedimentary environment, and the appearance of pyrite reflects the weakly reducing redox conditions of the sedimentary environment. The continuity of the sand body is relatively poor, and the carbonaceous and argillaceous layers form small interlayers, resulting in strong oil heterogeneity. The development of bright crystalline muscovite is also evidence of a typical delta front depositional environment. In summary, the data from the sub-oil-bearing Chang 8_2_ formation suggest that the study area during the depositional period of this unit was generally a shallow-water delta front sedimentary environment with weak oxidation and weak reduction with frequent changes in the subaqueous sedimentary environments. This view is also consistent with previous research results^54–56^.

**Figure 7:**
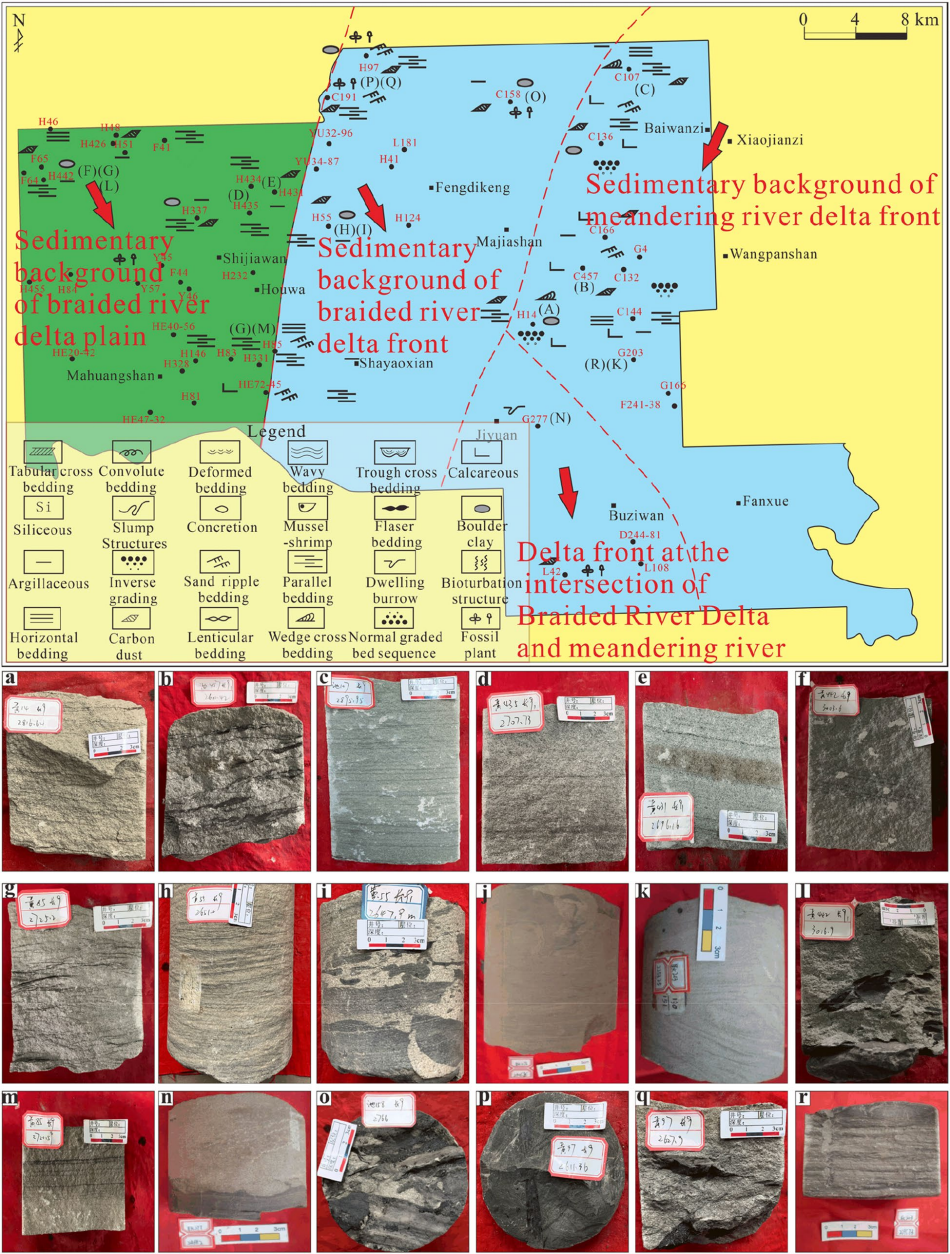
The planar distribution of sedimentary structures in the oil-bearing Chang 9 formation.

The oil-bearing Chang 9 formation reflects the characteristics of the transition from the delta plain to the front in the study area from west to east, which is well confirmed by the results of previous studies^3,11^. Although researchers have previously reached some understanding of the river delta sedimentary environment of the Chang 9 formation in the Jiyuan area^43,57^, there has been no detailed work on the distribution of sedimentary subfacies. This study revealed that the distribution of the Chang 9 sedimentary subfacies from west to east in the Jiyuan area exhibits certain zoning (Fig. 7).

In the braided river delta plains, carbon debris is relatively common, and plant fossil debris is also relatively abundant. In addition, mud and gravel adhesion can be seen in river sediments with strong hydrodynamics. In the braided river delta front, abundant sand-line bedding can be seen, and the mudstone is mainly grey‒green. The overall grain size of the sandstone is relatively coarse, the grains are rounded and sorted, and plant fossils are common. In addition, the carbon debris is obvious, reflecting the characteristics of weakly oxidized underwater sedimentation as a whole, and the sedimentary sequence is mostly dominated by a discontinuous positive rhythm. In the meandering river delta front, the mudstone is mostly dark, with a low content of impurities, a finer grain size, better sorting and rounding, common sand grain bedding, and more developed plant rhizomes. The presence of parallel bedding and gravel-sized mud clasts are sedimentary characteristics indicative of an underwater distributary channel. The anti-granularity phenomenon, which is an important indicator, is extremely obvious, usually representing the anti-rhythm and compound rhythm characteristics of estuary bar sedimentation. The water was relatively shallow, and the development of carbon chips can be seen occasionally.

### Classification of sedimentary facies

Three sedimentary microfacies are mainly developed in the shallow-water delta of the Chang 8_2_ formation, including underwater distributary channels, underwater natural levees and interdistributary bays. The underwater distributary channels are mainly composed of fine sandstone, the underwater natural levees are mainly composed of silty fine sandstone and siltstone, and the distributary bays are mainly composed of mudstone and silty mudstone. The underwater distributary channels are associated with sedimentary structures reflecting stronger hydrodynamic conditions, such as cross-bedding, parallel bedding and massive structures.

The sedimentary environment of the Chang 9 formation was mainly a river delta system. This environment included a braided river delta and a meandering river delta, but the main sedimentary microfacies are mainly composed of the following types. At the delta front, the sedimentary microfacies are mainly underwater distributary channels, underwater natural levees and interdistributary bays. In terms of sedimentary structures, the differences between the underwater distributary channels of braided rivers and those of meandering rivers have been described above.

In the delta plain subfacies, the study area mainly features a braided river delta system, including distributary channels, natural levees, crevasse splays, interdistributary depressions and peat swamps. The lithology of the distributary channels is mainly fine sandstone and a small amount of medium sandstone, with large cross-bedding and other sedimentary structures reflecting strong energy. The natural levees and crevasse fans exhibit reverse grain size sequences, and the lithology mainly includes siltstone and fine siltstone. The interdistributary depressions mainly feature mudstone and silty mudstone, with little difference in lithology from the underwater sedimentation of the delta front. The peat swamps were dominated by mudstone deposition, but the colour of the mudstone usually reflects an oxic sedimentary environment, and carbonization and other sedimentary structures can be seen.

Based on the facies evidence, it is believed that the development of the sedimentary system in the study area consisted of two parts. The transport and deposition of sediment from the Alxa ancient land to the northwest led to the development of a braided river delta sedimentary system in the western and central parts of the study area, while the transport and deposition of sediment from the Yinshan ancient land to the north led to the development of a meandering river delta sedimentary system in the eastern part of the study area. These sedimentary systems with different provenances formed a confluence in the southern part of the study area (Table 2).

**Table 2:** Composition and distribution of sedimentary facies types in the sub-oil-bearing Chang 82 formation and the oil-bearing Chang 9 formation.

Oil-bearing formation	Sedimentary facies	Sedimentary subfacies	Main sedimentary microfacies	Distribution location in the study area
Chang 8_2_	Shallow delta	Shallow delta front	Underwater distributary channel	Entire area
			Subaqueous levee	
			Distributary bay	
			Estuary bar	
			Sand sheet	
Chang 9	Braided river delta	Braided river delta front	Underwater distributary channel	Central part
			Subaqueous levee	
			Distributary bay	
		Braided river delta plain	Distributary channel (braided channel)	Western part
			Natural levee, rupture fan	
			Diversion depressions, peat swamps	
	Meandering river delta	Meandering river delta front	Underwater distributary channel	Eastern part
			Subaqueous levee	
			Estuary dam	
			Distributary bay	
	Confluence area	Delta front	Underwater distributary channel	Southern part
			Distributary bay	
			Subaqueous levee	
			Sand sheet	
			Estuary bar	

## Analysis of the hierarchical structure of architectural elements

In the Chang 9-Chang 8_2_ formations, from bottom to top, the development of sand bodies shows an overall declining trend as the lake level gradually rose. The delta plain sand body of the oil-bearing Chang 9 formation formed in the lower part of the accommodation space. Under the strong sediment supply, the sand bodies are frequently stacked vertically and alternately cut laterally. When the sand bodies of the delta front formed, the overall water level was still low, and the accommodation/sediment supply (A/S) ratio was relatively low. When the sub-oil-bearing Chang 8_2_ formed, the water level was relatively high, and the shallow delta front sedimentary pattern formed on a gentle basin slope, which caused the sand body to form a complicated internal structure under the control of various sedimentary factors.

### Sequence division basis

This paper mainly refers to the following aspects for the division of different levels of base level cycles: changes in the properties of a single petrophysical facies, low-energy sediments, river erosion surfaces, changes in cycles and superimposed patterns, changes in facies or facies combinations, changes in sedimentary discontinuities, and lake flooding at various levels. The long-term SQ2 cycle in which the oil-bearing Chang 8_2_–Chang 9 formations are located is bounded by the sequence boundaries SB2 and SB3. SB2 is the dividing line between the Chang 10 formation and the Chang 9 formation, and it can be observed directly at the Rui River outcrop. SB3 is a surface related to the transition between proliferation and degeneration within the Chang 8 oil-bearing formation. The logging and outcrop characteristics are similar to those of SB2^31^.

### Developmental characteristics of medium-term cycles

**Figure 8:**
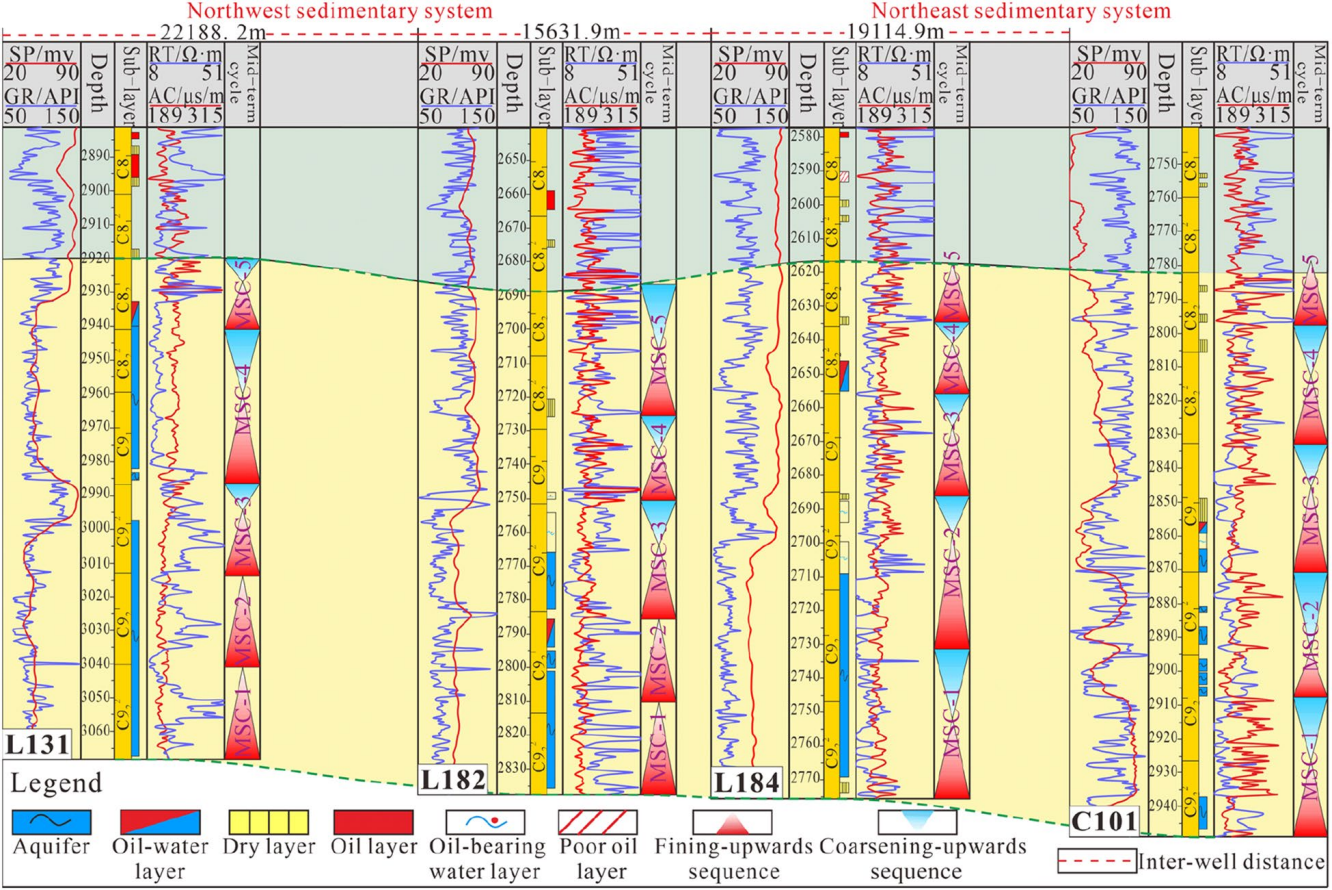
Identification and division of medium-term cycles in typical cored wells (Note: SP, GR, RT and AC are abbreviations of logging curve names. SP represents the spontaneous potential curve, GR represents the natural gamma curve, RT represents the resistivity curve, and AC represents the interval transit time curve. The medium-term cycles are abbreviated MSC-i).

In this study, 4 typical cored wells in different regions (Fig. 2c) were selected to carry out the division and identification of medium-term cycles (Fig. 8).

Affected by different sedimentary systems in the northwest and northeast, two types of medium-term cycle assemblages with different assemblage styles developed. In the northwest, both MSC-1 and MSC-2 are asymmetrical structures that deepen upwards (A1) and represent the distributary channels of the braided river delta plain with very small accommodation space. MSC-3, MSC-4, and MSC-5 are all symmetrical cyclic structures (C), reflecting the relatively complete stratum cycle process of several large-scale lacustrine regressions. MSC-1 to MSC-4 in the northeast are all symmetrical cyclic structures (C), reflecting the relatively large accommodation space in the sedimentary environment of the meandering river delta front. The relatively fine-grained mud and silt deposited during the lacustrine regression process were not completely eroded, so they are retained in the vertical sequence. On the other hand, MSC-5 is an upwardly deepening asymmetrical cycle (A2) under the background of deeper water and higher accommodation space, forming an undercompensated sedimentary environment with a lack of sediment supply.

### Short-term cyclic development characteristics

Based on the sedimentary microfacies and the identification of the medium-term cycle sequence interfaces, the short-term base level cycles are identified and divided according to the facies architectural relationship caused by the sedimentary genesis.

**Figure 9:**
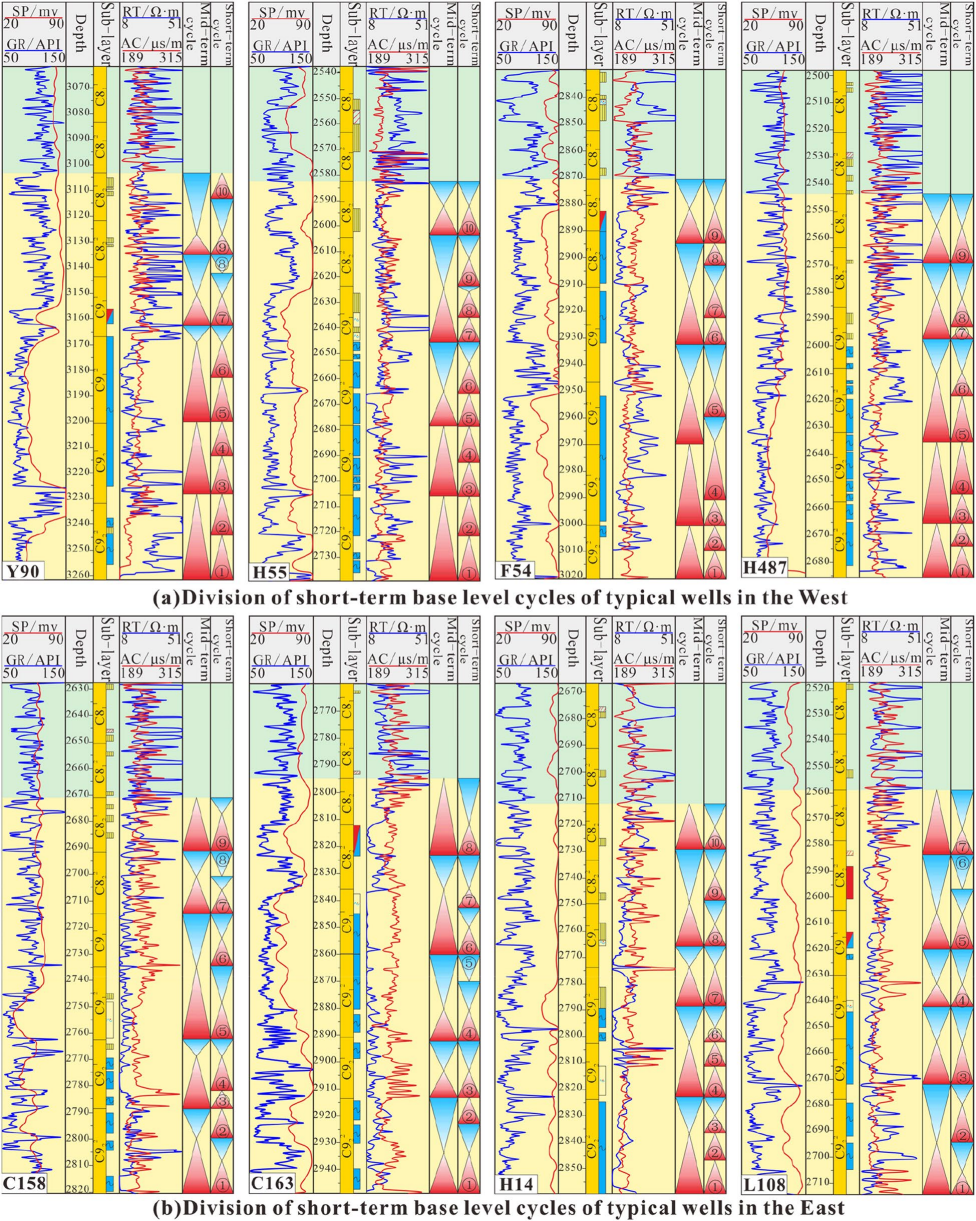
Results of short-term base level division in the study area (Note: SP, GR, RT and AC are abbreviations of logging curve names. SP represents the spontaneous potential curve, GR represents the natural gamma curve, RT represents the resistivity curve, and AC represents the interval transit time curve. Short-term cycles are represented by the symbols ①–⑩).

Eight cored wells with a high degree of penetration were selected (Fig. 2c). Based on the core descriptions and the mutual calibration of logging curves, combined with the regional characteristics of the sedimentary system, short-term base level cycle division and identification were carried out. The results for typical wells in the west show that due to changes in the sedimentary systems, the short-term cycles over a relatively large area were not isochronous (Fig. 9a). The medium-term cycles are divided into 9 or 10 short-term cycles. The short-term cycles in the lower part of the whole segment are mostly asymmetrical cycles that deepen upwards (A1). In the middle and upper parts, the cycles gradually become completely or incompletely symmetrical cycles that first deepen and then shallow upwards (C). The types of short-term cycles mainly include A1, A2, B1, C1, C2, and C3. The results for typical wells in the east show that the vertical distribution of short-term cycles is more variable than that in the west (Fig. 9b). Over a large area, there is no superposition of short-term cycles with significant isochronous characteristics that can be continuously compared. There are usually 7 to 10 short-term base level cycles in the study area, forming medium-term cycles. It is speculated that this is due to sedimentary transition and provenance intersection. From bottom to top, the whole section is mainly a fully or incompletely symmetrical cycle structure (C) that first becomes deeper and then becomes shallower. The lower part of the local area still retains an upwardly deepening asymmetrical cycle (A1). In addition, the symmetrical cyclic structure transforms from C1 to C2 and C3, and the short-term cyclic types include A1, B1, B2, C1, C2, and C3.

### Sequence filling process response

**Figure 10:**
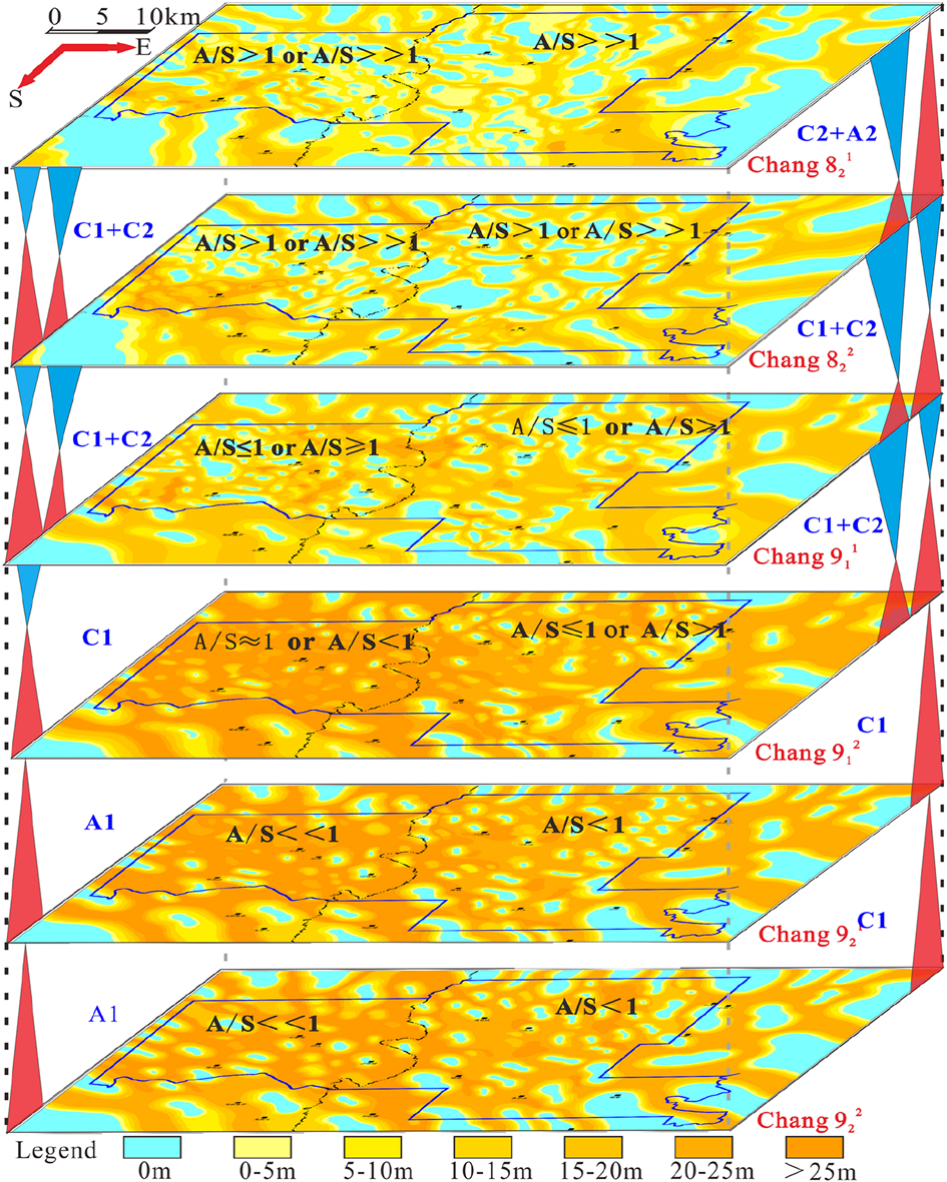
Sand body distribution responses to the dynamic process of sequence filling.

Changes in the accommodation space and sediment supply control the type and distribution pattern of the cyclic structure. These changes are also closely related to spatial changes in the sedimentary microfacies, and a corresponding dynamic response process is formed with sequence filling (Fig. 10). The transition from the oil-bearing Chang 9 formation to the sub-oil-bearing Chang 8_2_ formation reflect the process of an initial large increase and then a slight decrease in the water level during the depositional period. The Chang 9_2_^2^ and Chang 9_2_^1^ layers in the lower part of the oil-bearing Chang 9 formation were deposited under low accommodation space conditions (A/S <  < 1). In the braided river delta sedimentary system, the A1 type (upwardly deepening asymmetrical type) is more common in the delta plain, as evidenced by the vertical stacking style of the braided river channels. The sedimentary records of the ascending semicycle are preserved, and the descending semicycle is eroded, reflecting the frequent overlapping of channel sand bodies and active lateral migration.

The Chang 9_1_^2^ layer, mostly composed of incompletely symmetrical cycles dominated by ascending half cycles (C1), was deposited under relatively high accommodation space conditions (A/S ≈ 1 or A/S > 1), presenting transgressive systems tract characteristics. The ascending semicycle facies domain is larger than the descending semicycle facies domain, and it is common in sedimentary areas where underwater distributary channels and interdistributary bays alternately develop.

The Chang 9_1_^1^ layer is combined with the cycle structure of the upper Chang 8_2_^2^ layer and Chang 8_2_^1^ layer to form two symmetrical cycle structures (type C). The accommodation space increased significantly under the influence of lake flooding in the upper part of the Chang 9 reservoir group (A/S > 1 or A/S >  > 1). The cycle structure is mainly composed of an incomplete symmetrical type (C1) dominated by an ascending semicycle and a nearly complete symmetrical type (C2) with ascending and descending semicycles of nearly equal thickness. The sedimentary environment transformed into a large depositional area at the delta front. From bottom to top, the lower part of the underwater distributary channel sand bodies transition into thin layers of siltstone and mudstone deposited on overflow banks and then upward into silty fine sandstone at the front edge of the underwater distributary channels.

In the meandering river delta sedimentary system, the oil-bearing Chang 9 formation mainly developed in a delta front sedimentary environment. Therefore, the accommodation space was higher than that of the braided river delta sedimentary system (A/S ≤ 1 or A/S > 1), and thus a cyclic structure type C with initial deepening and subsequent shallowing developed. The lower part is mostly composed of the C1 type, and the proportion of the C2 type gradually increases upwards. In regard to the sub-oil-bearing Chang 8_2_ formation, a relatively high water level systems tract formed under the influence of lake flooding, and type A2 also occasionally manifested.

## Characterization of sand body architectural elements

### Facies architecture interface recognition

**Figure 11:**
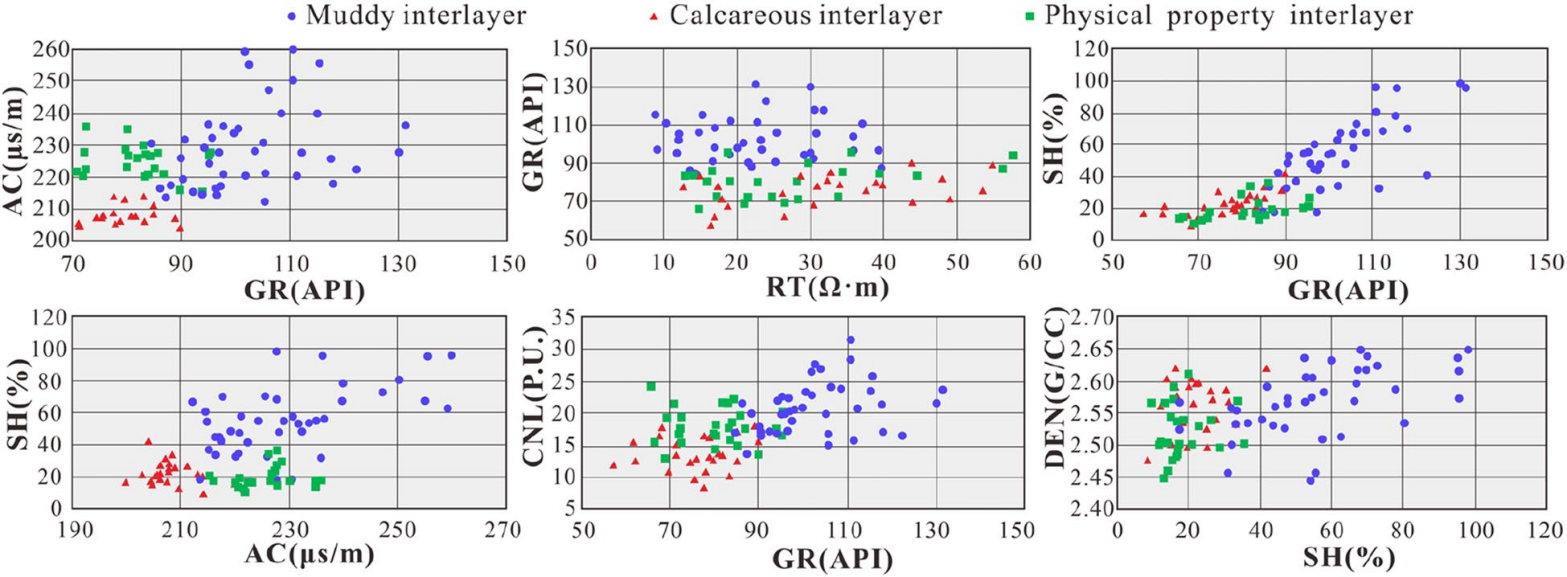
Intersection chart of electrical measurement curves of architectural interfaces between various facies.

The results show that discontinuities in sediments can cause sudden changes or weak returns in the GR and AC curves. The combination of lithological changes and the interpretation of oil-bearing sediments can assist in distinguishing the continuity of a sedimentary sequence (Fig. 11).

The GR and AC curve parameters have the ability to effectively identify architectural interfaces, and the GR–AC intersection diagram is an effective tool. The distribution of calcareous and muddy interlayers occupies the lower left and upper right areas, and the boundaries are GR = 90 API and AC = 214 μS/m. The AC values of interlayers with good physical properties are relatively high, mostly greater than 215 μS/m. The introduction of the SH curve reveals that the SH values of calcareous interlayers and interlayers with good physical properties are mostly less than 32% and that the SH value of muddy interlayers is higher than this threshold. The combination of the CNL and DEN curves suggests that the muddy and calcareous interlayers can be defined by CNL values greater than 16 and various DEN values. The calcareous interlayers are affected by carbonate cementation, and their density is relatively high, while the compensation neutron parameter is relatively low (Table 3).

**Table 3:** Electric identification parameters of facies architectural interlayers.

Facies architecture interlayer	SP	GR	RT	AC	SH	CNL	DEN
	mV	API	Ω m	μs/m	%	P.U	g/cm^3^
Muddy interlayer	7.89–95.8	84.58–131.41	8.83–39.75	212.29–259.82	17.22–98.12	13.64–31.45	2.44–2.65
Average	60.94	102.12	21.99	229.32	54.85	20.78	2.57
Calcareous interlayer	6.04–124.36	57.27–89.85	12.63–54.94	200.01–219.86	8.6–41.69	8.41–18.02	2.48–2.62
Average	50.84	76.13	31.30	208.23	22.13	13.51	2.57
Physical property interlayer	9.7–92.6	65.66–95.37	10.37–57.71	215.43–235.85	9.78–35.53	12.95–24.23	2.45–2.61
Average	64.18	79.69	26.70	224.86	17.75	17.73	2.52

### Lithofacies identification and classification of assemblage types

#### Lithofacies type

The lithofacies belong to Miall's 1st- to 2nd-level architectural elements. The lithofacies of the same sedimentary origin are superimposed to form a continuous lithofacies sequence, which is called a 3rd-level architectural element, corresponding to lateral accretion bodies or hyperplastic bodies. The combination of 3rd-level architectural elements can form monogenetic sand bodies, which are the main object of this study and correspond to ultrashort-term base level cycles. After drawing on the idea of the Miall lithofacies code compilation^42^, the authors identified the following 9 lithofacies types through core observations and petrological characteristics (Table 4).

**Table 4:** Nine lithofacies types based on core observations and petrological characteristics in the study area.

Lithofacies type	Code	Description
Massive bedding gravelly fine medium sandstone facies	Gm	The structural maturity is medium and low; Gravel content is relatively low; Scouring and filling structure can be seen in some cored wells; Strong hydrodynamic detention sedimentation at the bottom of the channel
Trough cross-bedding fine to medium sandstone facies	St	Grey, grey–white fine medium sandstone; Origin of sand bottom migration; Bottom or lower part of channel sediment; The development scale is related to the water velocity; Scale decreases upwards; The layer thickness of is small
Wedge cross-bedding fine medium sandstone facies	Sl	Greyish white fine sandstone; Staggered and wedge-shaped interlayer interface; Affected by lake waves in the delta front; Low angle oblique layer; There may be a change in flow direction
Plate cross-bedding fine medium sandstone facies	Sp	Greyish white fine sandstone; High angle and low angle undercut and downward truncation; The scale of strata changes greatly; Downstream accretion of longitudinal sand dam
Parallel bedding fine medium sandstone facies	Sh	Greyish white fine medium sandstone; Upper part of river channel; Wide gentle shallow-water delta environment; Vulnerable to cutting; relatively small thickness of strata; High flow regime; Forms a flat bed sand bottom
Sand bedding silty fine sandstone facies	Fr	Very fine sand and siltstone; Upper part of sedimentary sequence small flowing sand grain; The thickness of single layer is thin; Developed in abandoned channel or embankment deposits
Wavy bedding siltstone/argillaceous siltstone facies	Fsc	Grey/greyish green thin siltstone mudstone interbedding; The laminae are wavy and continuously distributed; Sandstone and mudstone are staggered; Developed in a relatively low energy environment; Fine particle size in overflow sedimentation or underwater natural embankment
Horizontal bedding silty mudstone/mudstone facies	Fl	Dark grey, grey–black siltstone and mudstone; Thin layer interface and distributed in a nearly horizontal manner; In abandoned channels or bank deposits; The energy decreases gradually with the flow across the bank
Massive bedding mudstone/silty mudstone facies	Fm	Dark grey, grey–black and black mudstone; Overbank deposits/abandoned channel deposits; Massive structure; The thickness of single layer is thin; Intercalation in composite channel; Poor stability and easy to be eroded; Irregular spatial form

#### Lithofacies association types and characteristics

By identifying the lithofacies sequence of a large number of cored wells, using core positioning technology, introducing the measured porosity and making it correspond to the acoustic time curve, and deriving the corresponding section of electrical measurement data, the petrologic-electric calibration process can be realized. The various lithofacies association (FA) types identified at this scale can be used as large-scale bottom shapes in the river delta sedimentary system, corresponding to architectural elements such as CH, SB, and OF in the river facies^46^.

**Figure 12:**
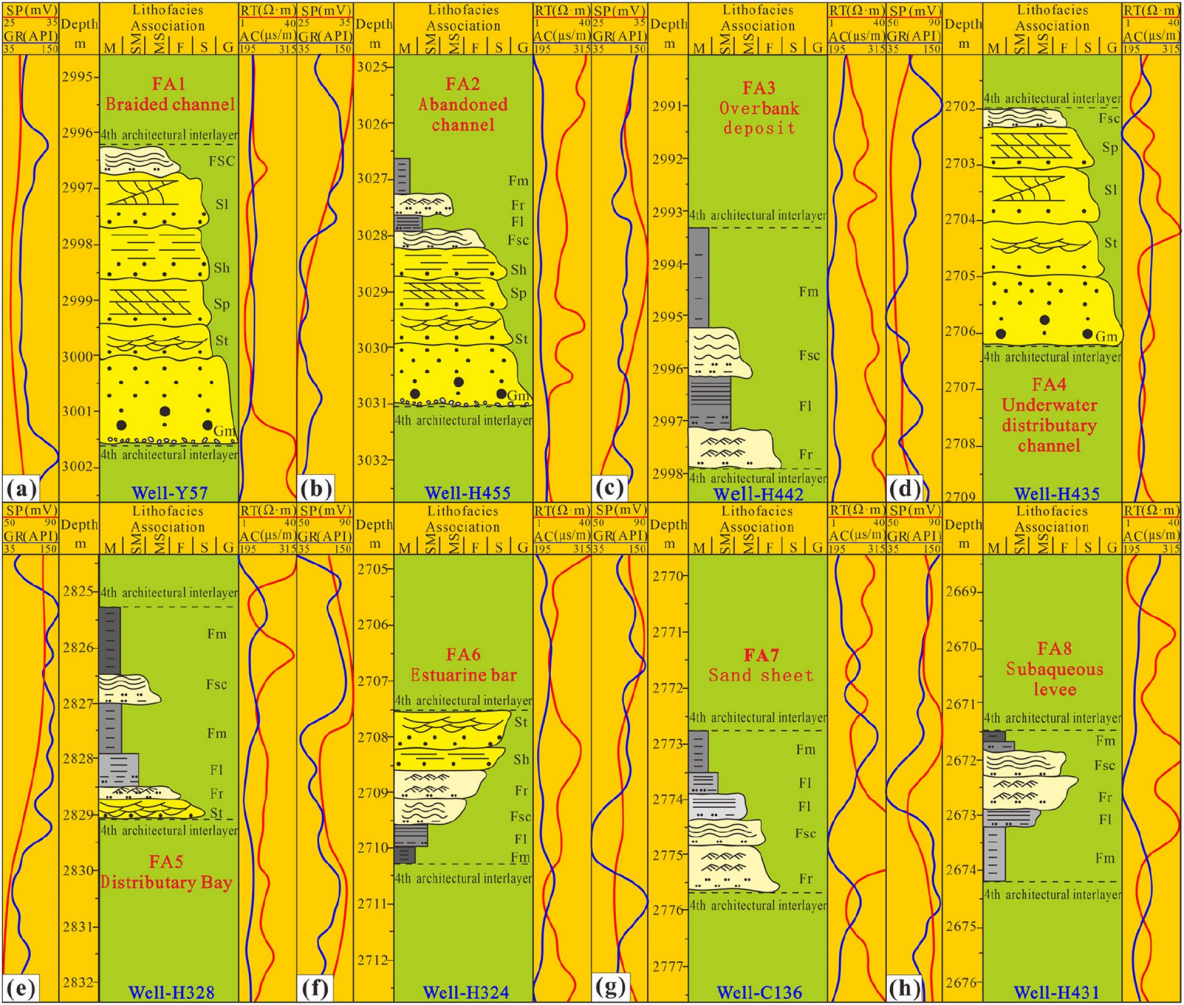
Eight FA types (architectural elements at the single sand body level) (Note: SP, GR, RT, AC are abbreviations of logging curve types and are consistent with the meanings in the above figures).

In the sub-oil-bearing Chang 8_2_ formation and the oil-bearing Chang 9 formation, 8 kinds of common structural elements are identified. The types of architectural elements (this paper refers to them as FAs) produced in the braided river delta sedimentary system in the northwest and the meandering river delta sedimentary system in the northeast are different (Fig. 12).

FA1 (Braided channel): As the skeletal architectural element of the braided river delta plain environment of the Chang 9 oil layer group in the west, this FA sequence is approximately Gm-St-Sp-Sh-Sl-Fsc (Fig. 12a). There are many types of bedding, mainly cross-bedding, reflecting strong hydrodynamic forces and an abundant sediment supply. The architectural elements of braided channels and alluvial plains together constitute the depositional environment of a braided river delta plain. The braided channels are characterized by high width-to-thickness ratios and high sand-to-soil ratios. The braided channels form wide flat sandstone belts with scouring surfaces at the bottom. The sequence stacking combined with glutenite can form a composite lens of horizontal and vertical sand bars or complexus. Generally, braided river channels are more commonly vertically stacked, forming a thick composite channel unit as a whole, with strong lateral continuity, and often interweaving with other braided channels.

FA2 (Abandoned channel): First, a river channel formed and was then abandoned. Later, it was filled with mud. From bottom to top, the particle size becomes finer, and the bedding scale is gradually reduced, reflecting the gradual decrease in hydrodynamic force. The channel-filling deposits are very obvious, and the sequence of the lithofacies combination is approximately Gm-St-Sp-Sh-Fsc-Fl-Fr-Fm (Fig. 12b). The overall shape is convex and flat on the top of the lens, converging and thinning to both sides, gradually pinching out due to the influence of the abandonment of channels, with relatively little overlap with other channels. The sand body is still the main body, and the fine-grained deposits in the upper part are relatively small. Due to the relatively low degree of compositing, it is easy to judge and identify when a single channel divides.

FA3 (Overbank deposit): Overbank deposits are one of the important configuration elements in the sedimentary environment of the braided river delta plain of the oil-bearing Chang 9 formation in the west, and the hydrodynamic force decreases upwards until it is close to the hydrostatic environment. The sequence of lithofacies combination is approximately Fr-Fl-Fsc-Fm (Fig. 12c). Affected by the migration of braided rivers, there are large changes in width. When the flood season came, the water overflowed over the channel, and both sides of the channel began to receive fine-grained sediments. These areas can develop into swamps in humid environments and can act as sites for coal accumulation. Occasionally, a small amount of sand in the form of small temporary channels is deposited in local overbank environments.

FA4 (Underwater distributary channel): In the eastern and western parts of the study area, the delta front environment of the oil-bearing Chang 9 formation and the shallow-water delta front environment of the sub-oil-bearing Chang 8_2_ formation occur frequently and are skeletal architectural elements. The lithofacies sequence is approximately Gm-St-Sl-Sp-Fsc (Fig. 12d). The hydrodynamic force gradually decreases upwards, and the deposits are lenticular in cross-section, with short lateral extents and rapid pinch-outs. In the braided river delta system, due to frequent channel migration, other channels are truncated laterally. At the same time, channels become vertically superposed with other channels. After several lens bodies are superimposed, a thicker underwater distributary channel composite sand body can be formed. In the meandering river delta sedimentary system, an underwater distributary channel has strong stability, and the combination of muddy sediments forms a pattern similar to the dual structure. Underwater distributary channels are also well developed in the sedimentary environment of the shallow-water delta front, but the degree of vertical composite overlap in this environment is further reduced, and they are mostly developed in combination with other architectural elements.

FA5 (Distributary bay): This architectural element is the fine-grained sediments developed in the underwater distributary channels, and the hydrodynamic force is relatively low. The lithofacies combination sequence is approximately St-Fr-Fl-Fm-Fsc-Fm (Fig. 12e), but this sequence is not completely fixed. The cross-sectional morphology is dominated by clay interlayers or thin lenticular shapes, and biogenic sedimentary structures can be seen in parts deposited in shallow water. This element usually transitions into sandy sedimentary facies and is the successor product of the lateral gradual change in the river channel. When the diversion of underwater distributary channels is active, the fine-grained deposits are prone to erosion and damage to varying degrees, and the geometric dimensions are difficult to determine. The overall hydrodynamic force is extremely weak, mostly associated with underwater natural dikes and sand sheets, which are produced in the upper part of the channel.

FA6 (Estuarine bar): This architectural element represents another skeleton configuration element in delta front sedimentation, and it is developed at the end of an estuary associated with an underwater distributary channel and at the side edge of an underwater distributary channel. In profile, this element has a flat bottom and a convex top, and the planar shape is fan-shaped. The lithofacies sequence is approximately Fm-Fl-Fsc-Fr-Sh-St (Fig. 12f). This element is an active sedimentary body in an estuary, and the main hydrodynamic conditions are dictated by the bottom plane jet of the density current. Usually, due to the combined action of rivers and lakes, estuarine bars are subject to repeated erosion and sorting. The well-sorted sandy sediments are kept intact and exhibit groove-like cross-bedding, and the layer thickness is not large. The development of estuary bars is closely related to the sedimentary environment, and they develop on a large scale in meandering river deltas. However, in braided river delta and shallow-water delta environments, they are impacted by river migration and the shape of the lake basin bottom and are susceptible to erosion, poor preservation, and limited scale.

FA7 (Sand sheet): This type is the deposit at the delta front edge, and the sequence of lithofacies is approximately Fr-Fsc-Fl-Fm (Fig. 12g). Through scouring and winnowing by waves and shore currents, sand sheets can develop widely. The sedimentary structure is similar to that of the estuarine bar, and cross-bedding can be seen. This sedimentary microfacies type reflects transformation from a constructive delta to a destructive delta. The study area was a delta front sedimentary environment with a strong constructive effect. Sheet sand development was limited, and the deposits are usually interbedded with mud.

FA8 (Subaqueous levee): This element is composed of sand ridges on both sides of an underwater distributary channel, and the lithofacies sequence is approximately Fm-Fl-Fr-Fsc-Fm (Fig. 12h). Subaqueous levees are mostly associated with underwater distributary channels and form a lithofacies sequence that is relatively stable at the front of a meandering river delta. However, in the environment of a braided river delta front with frequent river migration, development of this architectural element is restricted, and it is usually destroyed by the lateral cutting and erosion of diverted underwater distributary channels.

### Combination characteristics and spatial distribution patterns of sand body architectural elements

#### Types and characteristics of the vertical combination of architectural elements

Based on the identification of sedimentary structure divisions and architectural elements, the oil-bearing Chang 9 formation in the study area is roughly divided into the braided river delta plain in the west, the front transition area of the braided river delta plain in the middle, and the meandering river delta front in the east. In addition, the confluence area of the delta front is in the southeast. According to the analysis of sedimentary characteristics and the distribution of sedimentary structures, the sub-oil-bearing Chang 8_2_ formation is divided into eastern and western areas.

1. Vertical assemblage types and characteristics of the architectural elements of the Chang 9 formation.

**Figure 13:**
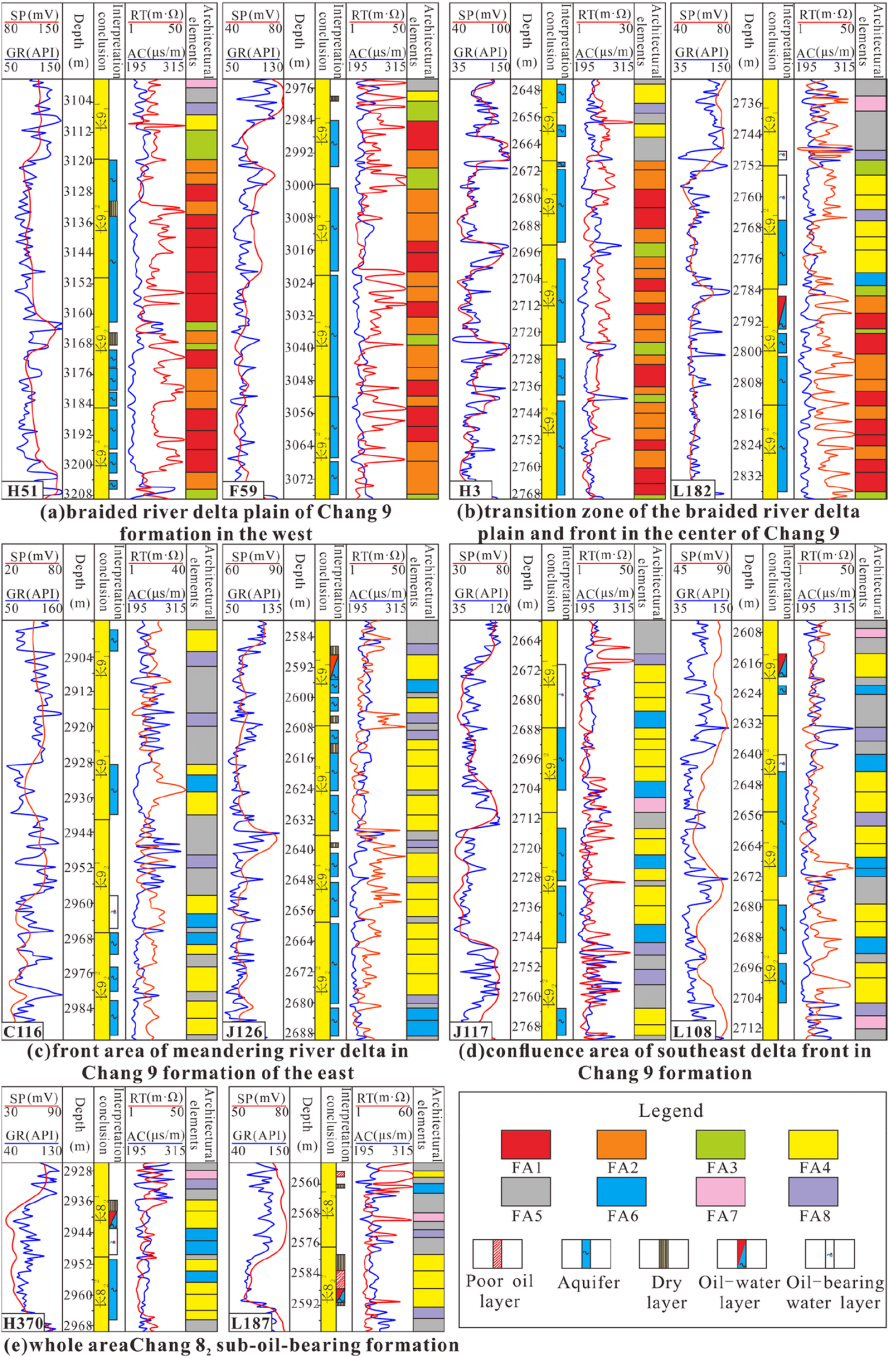
Vertical combination characteristics of architectural elements (Note: SP, GR, RT, AC are abbreviations of logging curve types and are consistent with the meanings in the above figures).

In the lower and middle-upper parts, typical braided river delta plain sedimentary sand bodies developed in the Chang 9_2_^2^, Chang 9_2_^1^, and Chang 9_1_^2^ layers (Fig. 13a). The main architectural elements include FA1, FA2, and FA3. Among them, two types of architectural elements, braided channels and abandoned channels, are frequently superimposed and combined to form composite braided distributary channels. A channel complex usually consists of 3–5 FA1 and FA2 deposits. During the intermittent period of river channel deposition, FA3 was deposited across the bank, and the whole section is dominated by sandy deposits from bottom to top, with a very high sand–silt ratio. In the upper Chang 9_1_^1^ layer, the plain sedimentary environment disappeared and was replaced by a braided river delta front environment. The hydrodynamic force suddenly decreased, resulting in the significant deterioration of the development degree of the sand bodies. It is speculated that the environment transitioned from distributary channels to a more lacustrine environment. FA4, FA5, and FA8 are common, but FA7 is less developed, and FA6 is not developed. In Well H51, FA1 is more developed than FA2, and 3–5 vertically superimposed architectural elements of FA1 can be seen, reflecting the extremely strong hydrodynamic force. In Well F59, FA2 accounts for significantly more than FA1. It is speculated that because the well is located relatively far from the provenance, the hydrodynamic force was lower, causing more braided channels to transform into abandoned channels, and the degree of vertical overlap decreases slightly.

In the lower and middle-upper parts, the Chang 9_2_^2^ and Chang 9_2_^1^ sublayers are still dominated by braided river delta plain deposits (Fig. 13b). FA1, FA2, and FA3 can be seen, and the sub-oil-bearing Chang 9_1_ formation gradually moved from the plain to the front edge. Well H3 is relatively close to the provenance, and the sedimentary characteristics of the plain are more well preserved, while the sedimentary characteristics of the front are more prominent in Well L182. In the sedimentary part of the braided river delta plain, the vertical combination of architectural elements is still mainly the composite superposition of FA1 and FA2. However, due to the migration of sedimentary areas to the east, the vertical superposition of FA1 is reduced, and the rest of the sandy fill is composed of FA2. In the braided river delta front deposits, FA4 overlaps significantly, and 2–3 FA4 units can combine to form a set of underwater distributary channel composite sand bodies. FA5, FA7, and FA8 are also common, while FA6 is less developed, which is related to the restricted development of estuarine bars.

The development and combination style of architectural elements in the east is significantly different from that in the braided river delta in the west (Fig. 13c). Well C116 is a typical architectural element combination mode of meandering river delta front sedimentation. FA4 appears alone or overlaps with other FA4 units and FA6, but the underwater distributary channels are relatively thin. The combination of FA5 and FA8 constitutes the part of silty and argillaceous sedimentation. The location of Well J126 is relatively close to the western system. It is speculated that the sediments represent a mixture of braided underwater distributary channel sand bodies. The submerged distributary channels may be composed of 2–4 superimposed FA4 units. Different from the estuary bar combination of Well C116, FA6 is rare in the development section of the FA4 superimposed combination in Well J126, which mostly features a large set of composite underwater distributary channel sand bodies. It is speculated that this is due to scouring and erosion by high-velocity flows and weak preservation. FA7 is relatively rare, indicating that the meandering river delta did not receive extensive sand inputs in the study area.

The vertical combination of architectural elements of the oil-bearing Chang 9 formation in the confluence area of the southeastern delta front is not much different from the normal delta front sedimentary characteristics. FA4, FA5, FA6, FA7, and FA8 are all observed, with FA4 being mainly stacked vertically (Fig. 13d). In addition, FA4 and FA5 are combined. Well J117 is located near the front confluence area, so sandy deposits are relatively well developed, while Well L108 is located at the far end of the front confluence area, and sandy deposits are weakly developed. This pattern is almost the same as that of the volume content of argillaceous deposits and has the characteristics of a dual structure. The estuary bar develop is significant, indicating that the hydrodynamic intensity in the sedimentary environment was lower than that in the previous areas, resulting in estuary bars directly overlying underwater distributary channels.

(2) Vertical combination types and characteristics of architectural elements of the Chang 8_2_ formation.

The Chang 8_2_ formation comprises shallow-water delta deposits. During this depositional period, the water level rose sharply, resulting in no significant difference in deposition between the east and the west of the Chang 8_2_ formation, thus forming the shallow-water delta front deposition pattern across the whole region. Therefore, Wells H370 (west) and L187 (east) were selected from the two main sand belts of the Chang 8_2_ formation for analysis of the vertical combination types and characteristics of the architectural elements (Fig. 13e). FA4 vertically overlaps small layers in Chang 8_2_^2^. The combination of FA4 and FA6 formed a composite underwater distributary channel sand body. FA5, FA7, and FA8 are also visible and are composed of silty and argillaceous sediments.

#### Prediction of the geometry of the river delta system

The geometric parameters of the four types of skeletal architectural elements are predicted (Table 5).

**Table 5:** Geometric parameters of the four main skeletal architectural elements.

Code	Architectural element	Thickness (m)	Width (m)	The ratio of width to thickness	Fourth-level interface inclination (°)
FA1	Braided channel	4.8–8.3	148.2–356.5	28.2–44.5	2.5
FA2	Abandoned channel	3.4–5.8	105.6–286.4	18.5–27.1	3.0
FA4	Underwater distributary channel	4.2–7.8	92.5–252.6	12.5–24.6	1.5
FA6	Estuarine bar	1.9–4.7	46.0–82.7	9.8–17.6	2.0

The braided channel is closer to the previous survey, with a larger width, ranging from 28.2 to 44.5. The thickness of the structural elements of the abandoned river channel is less than that of previous predictions, the extension width is also slightly reduced, and the width-to-thickness ratio is between 18.5 and 27.1. The width-to-thickness ratios of the architectural elements of the underwater distributary channel and the estuary bar in the delta front microfacies are slightly larger than those of previous predictions. The architectural element width-to-thickness ratio of the underwater distributary channel is between 12.5 and 24.6, and the architectural element width-to-thickness ratio of the estuary bar is between 9.8 and 17.6. The other types of architectural elements are mostly silty and muddy deposits, which do not match the prediction model of the skeletal elements, so they are not included in the estimation.

#### Types and characteristics of the lateral combination of architectural elements

**Figure 14:**
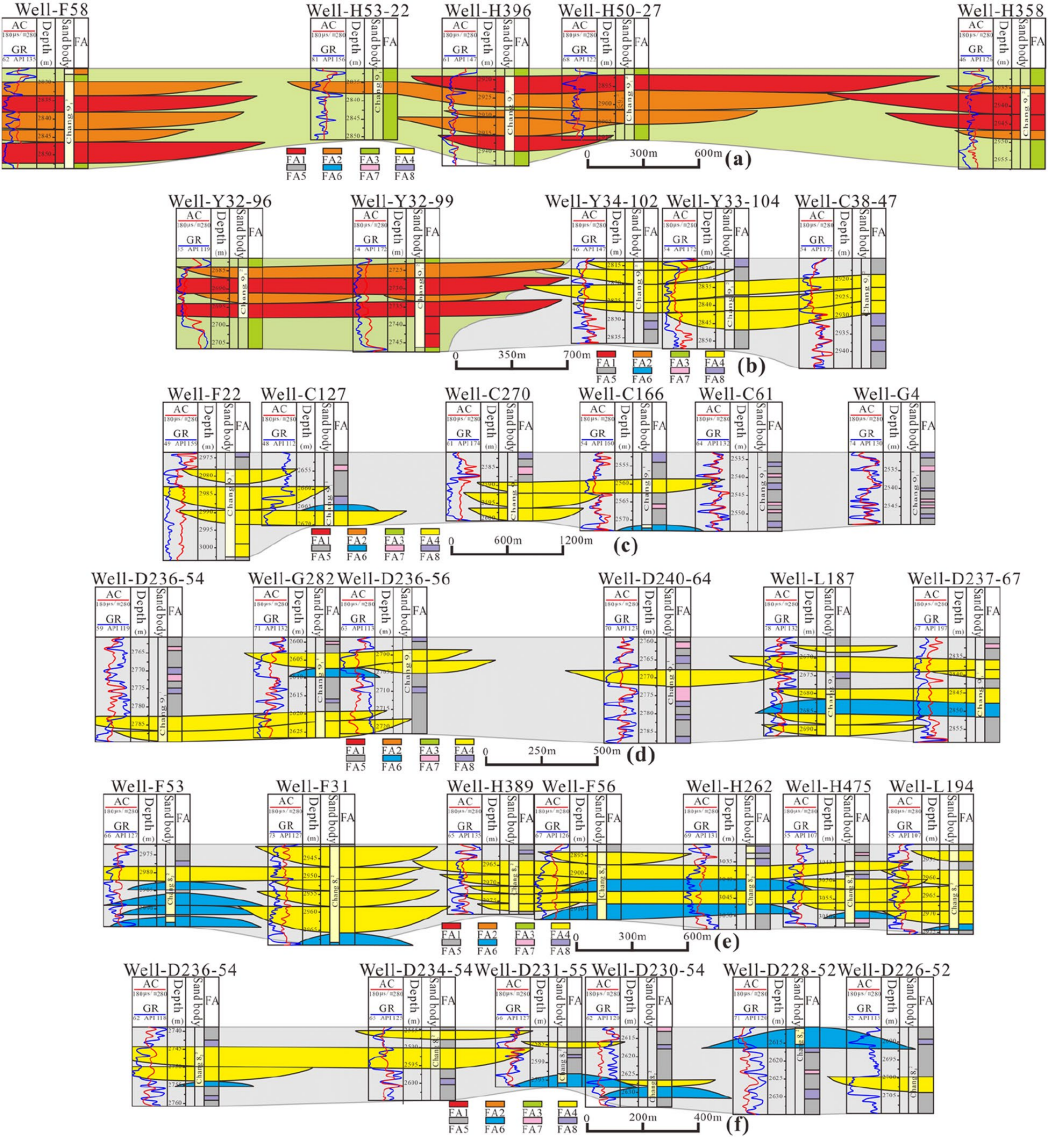
Types and characteristics of the lateral combination of architectural elements.

The analysis of cross-sections selected in different regions of the study area reveals that there is a difference in the lateral assemblage of the architectural elements between the oil-bearing Chang 9 formation and the sub-oil-bearing Chang 8_2_ formation, which is caused by the influence of sedimentary genesis (Fig. 2c, 14).

In the lateral combination of architectural elements in the braided river delta plain area of the western oil-bearing Chang 9 formation, in section AA' (Fig. 14a), the Chang 9_1_^2^ layer is characterized by significant FA1 and FA2 architecture that jointly form the braided river delta plain composite distributary channel sand body, and the four wells are characterized by extremely thick deposits. When every single architectural element is combined laterally, it can be seen that the width of the lateral complex is relatively large, and the abandoned channel FA2 is vulnerable to downcutting by FA1 in the upper part and lateral erosion by FA1 on the right. After the vertical and lateral architectural elements are integrated to form a large lens-shaped five-level architectural element, it can be seen that the lateral extension can reach 3–5 well distances, and the width extends 1–2 km.

In the BB' section of the lateral combination of the front edge transition area of the braided river delta plain in the middle of Chang 9 (Fig. 14b), FA1 and FA2 are still relatively well developed in the sedimentary environment of the left plain. The lateral complex of FA1 architectural elements frequently cuts down into FA2, resulting in serious abandonment of FA2 sandwiched in the middle and significant reduction of the geometric shape. The overall scale of the composite distributary channel sand body (5th-level architectural elements) formed by the integration of the two is still considerable, with an extension width of approximately 1.5 km and strong continuity. In the sedimentary environment of the right delta front, the thickness of the composite sand body (level 5) formed by 4th-level architectural elements decreases significantly, and the geometric shape has progradation characteristics. Three to four FA4 units form composites laterally, and the extension width decreases slightly. It should be noted that the transformation process from FA1 to FA4 can be identified in some other transition zone sections. There is a spatial form of FA1 cutting laterally into FA4, and the two types of architectural elements can form a composite 5th-level architectural element.

The architectural elements of the meandering river delta front area of the oil-bearing Chang 9 formation in the east are laterally combined in the CC' profile (Fig. 14c). Since there are few development wells in this area, the profile is formed from exploration wells over a large horizontal scale, and the results reflect the overall characteristics of relatively poor sand body development in the sedimentary environment of the meandering river in the east. The underwater distributary channel architectural element FA4 is relatively well developed, but the lateral recombination degree is generally poor, and the extension distance ranges from 1.2 to 1.8 km. Vertically, the FA4 architectural elements overlap frequently, and a composite underwater distributary channel sand body with a thickness of approximately 20 m can be formed under the combination of 3–4 FA4 units (5th-level architectural elements).

In the lateral combination DD' profile of architectural elements in the delta front confluence area in the southeastern portion of the oil-bearing Chang 9 formation (Fig. 14d), the lateral composite width of the FA4 architectural elements decreases significantly to below 0.6–1.2 km, which is caused by the weakening of ponding power after the rise of the water body in the lake basin. Single FA4 units in combination with FA7, FA8, and FA5 have branch channel characteristics, and FA6 architectural elements appear above and below FA4. In Well G282, the scale of FA6 is reduced sharply due to the downcutting of the overlying FA4. FA6 in Well L187 overlaps FA4 in the upper part of D237-67, but the thickness of the FA6 at D237-67 is reduced due to the downcutting of the overlying FA4.

The lateral combination of architectural elements in the western portion of the sub-oil-bearing Chang 8_2_ formation EE’ section is one of the main development blocks of Chang 8_2_. It is located at the intersection of the sand belts, and the sand bodies are connected (Fig. 14e). It can be seen that FA4 and FA6 are widely developed. There are 5 FA4 units vertically stacked in Wells F31 and H389, with a thickness of approximately 25 m. The lateral complex of architectural elements has a large extension width of more than 2 km, covering 4–5 well spacings. The process of the lateral recombination of FA4 and FA6 has the characteristics of ‘filling’ of architectural elements. The lateral complexes of FA6 in Wells F56 and H262 formed between the large lenses formed by FA4 recombination, which are locally scoured and eroded, reducing the geometric shape.

The lateral combination FF' profile of architectural elements in the sub-oil-bearing Chang 8_2_ formation in the east is also located in the main development block of Chang 8_2_ but featured a further increase in transport distance and a large coverage depth of the lake in the basin. The lateral continuity of FA4 architectural elements is significantly reduced, and the degree of vertical overlap is also attenuated (composed of 1 to 2 FA4 units) (Fig. 14f). Individual FA4 units show the characteristics of terminal branch channels, and the geometric shape is significantly reduced, as evidenced by the FA4 units in D231-55 and D230-54. Due to the weakening development of the underwater distributary channels, the development of the estuarine bar was enhanced, and FA6 develops into a complete ‘convex top and flat bottom’ shape. In this environment, the number of FA5, FA7, and FA8 architectural elements increases, reflecting the further maturity of the sedimentary development of the river-controlled delta front.

### Quantitative logging identification of sand body architectural elements based on genetic analysis

The electrometric curve characteristics of typical configuration elements have been analysed, and the results are as follows (Table 6).

**Table 6:** Logging curve parameter statistics of each architectural element (sample number: 239).

Type	SP	GR	RT	AC	DEN	PE	SH	Number of samples
FA1	76.84	79.89	24.64	222.06	2.46	3.12	14.93	32
FA2	75.05	81.32	23.26	223.55	2.48	3.12	16.77	39
FA3	85.47	107.00	54.52	228.22	2.58	3.36	53.57	19
FA4	54.89	69.07	21.26	222.78	2.50	3.11	16.21	63
FA5	67.32	103.67	17.16	240.15	2.58	3.39	64.08	41
FA6	58.84	78.45	23.55	220.26	2.52	3.25	23.34	15
FA7	64.11	86.52	23.19	233.31	2.59	3.33	43.01	9
FA8	68.86	86.52	22.56	230.44	2.58	3.26	42.35	21

The above data are the thickness-weighted average values of the logging parameters of the main target horizon. *SP* is the spontaneous potential logging curve, *GR* is the natural gamma logging curve, *RT* is the resistivity logging curve, *AC* is the acoustic transit time logging curve, *DEN* is the density logging curve, *PE* is the photoelectric cross-section index; *SH* is the shale content.

The characteristic value of the typical discriminant function is 4.505, and the typical correlation is 90.5%, so the discriminant analysis is effective. Finally, the classification function coefficient table (Table 7) is obtained, and the discriminant formula for each architectural element can be established based on the table. The expression method is as follows:

$${\text{F}} = {\text{SP}} \times {\text{K}}1 + {\text{GR}} \times {\text{K}}2 + {\text{RT}} \times {\text{K}}3 + {\text{AC}} \times {\text{K}}4 + {\text{DEN}} \times {\text{K}}5 + {\text{PE}} \times {\text{K}}6 + {\text{SH}} \times {\text{K}}7 + {\text{B}}$$

**Table 7:** Classification function coefficients.

Logging	Discriminant coefficient	FA1	FA2	FA3	FA4	FA5	FA6	FA7	FA8
SP	K1	− 0.009	− 0.017	− 0.019	− 0.045	− 0.043	− 0.053	− 0.034	− 0.022
GR	K2	0.506	0.511	0.494	0.379	0.353	0.448	0.327	0.325
RT	K3	0.105	0.104	0.178	0.096	0.128	0.103	0.119	0.115
AC	K4	7.527	7.556	7.432	7.587	7.524	7.483	7.635	7.578
DEN	K5	1296.871	1303.14	1298.671	1314.433	1305.243	1307.401	1327.403	1321.034
PE	K6	1.408	1.369	2.878	0.808	2.486	1.585	1.788	1.638
SH	K7	− 5.806	− 5.813	− 5.312	− 5.745	− 5.16	− 5.658	− 5.457	− 5.432
(Constant)	B	− 2414.946	− 2436.401	− 2420.894	− 2458.947	− 2448.555	− 2427.286	− 2511.805	− 2483.199

**Figure 15:**
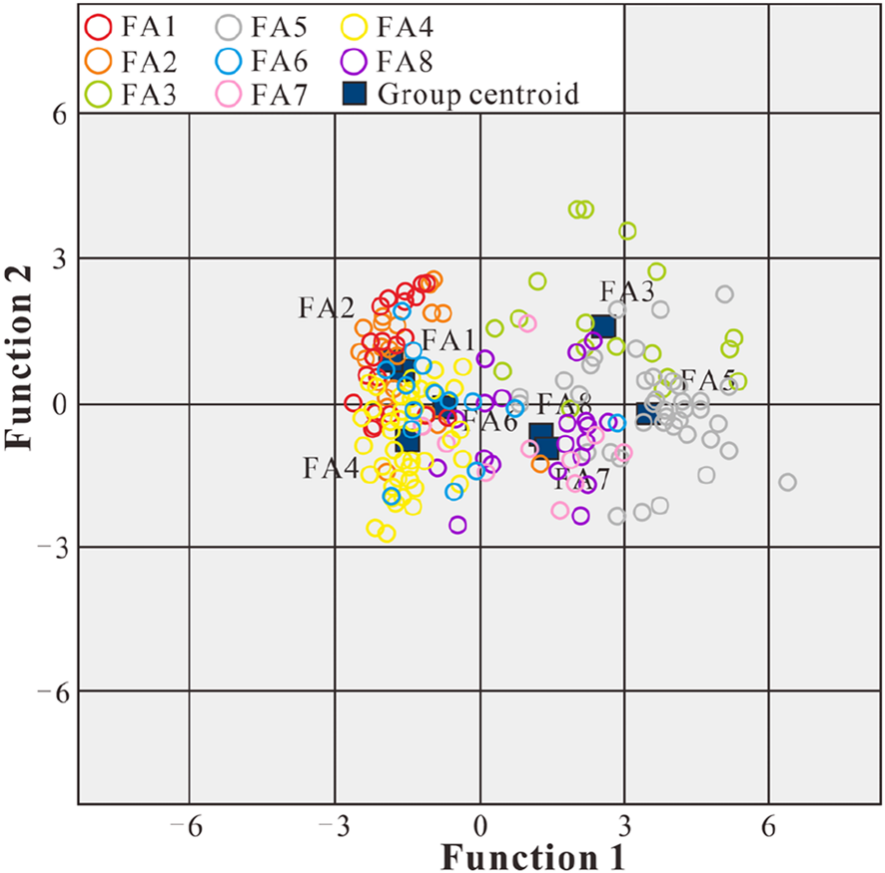
Typical discriminant function domain diagram.

The typical discriminant function domain diagram shows that the various architectural elements after each discriminant analysis have significant zoning characteristics (Fig. 15). The cluster centroids of FA1 and FA2 are relatively close, which is probably caused by the similar facies architecture characteristics of the braided channels and the abandoned channels in the braided river delta plain in the study area. In addition, due to the similar lithologic compositions of the two types of architectural elements, the ability to discriminate between them based on PE is reduced. Moreover, because the sand content in the two types of architectural elements is much higher than the mud content, the discrimination effects of GR, SP and SH related to the mud content are also significantly reduced. Thus, the discrimination effects are relatively insignificant.

In a braided river delta plain environment, the large set of 5th-level composite distributary channel architectural elements are mostly formed by the composite superimposition of the 4th-level FA1 and FA2, which has a small impact on the overall architectural collocation. The centroids of other architectural elements are far apart, indicating effective discrimination analysis. Based on the final classification statistical data verification, 54.4% of the case classification results are correct, and most of the errors are caused by the mutual misjudgment of FA1 and FA2 due to the closeness of their centroids, so the impact on the overall discriminant analysis is small.

### The spatial distribution characteristics of architectural elements

**Figure 16:**
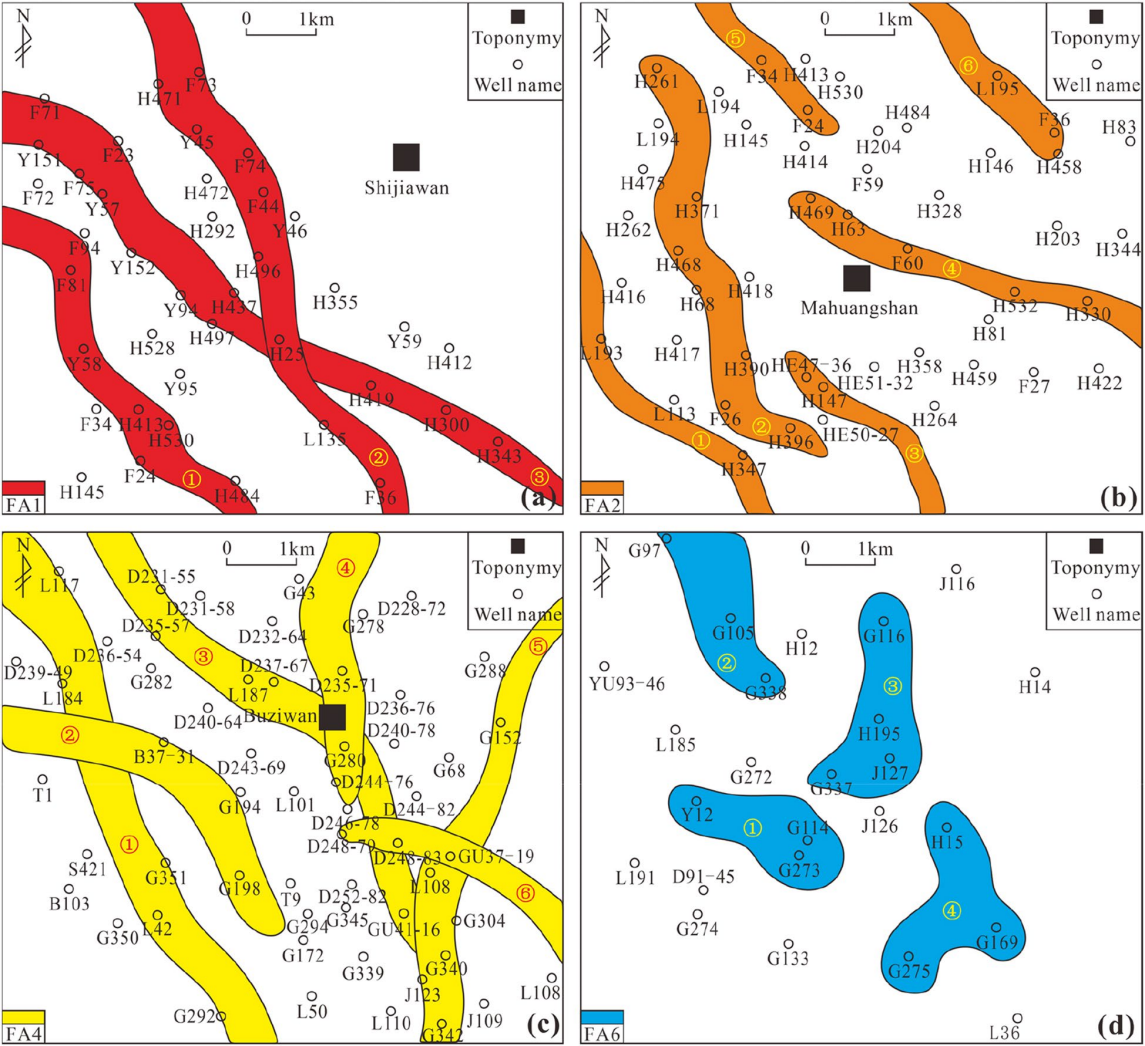
The local horizontal distribution of skeletal architectural elements.

After the architectural elements were identified by logging, four blocks were selected in the study area to study the spatial distribution of the four types of skeletal elements FA1, FA2, FA4, and FA6 (Fig. 16). Since the thickness of each architectural element is relatively thin, the isochronous single-cause architectural element of the same period is selected for spatial analysis.

The horizontal extension distance of FA1 architectural elements is large, and intersections are observed in individual wells and represent the lateral cutting of these elements. The FA2 architectural element is an abandoned channel, with a significantly shorter extension distance than FA1, relatively less lateral contact, and an intermittent horizontal distribution. It is speculated that this is caused by the lateral cutting of the FA1 architectural element. In the horizontal distribution of FA4 architectural elements, it can be seen that the extension distance is also large. At the same time, there are certain lateral contacts between the two architectural elements deposited in the same period, which can form a short intersection. The horizontal extension pattern presents a certain instability and can exhibit sudden discontinuities. The FA6 architectural element is an estuary bar, and its horizontal distribution is related to (underwater) distributary channel deposition. Generally, a small block or sheet distribution shape can form on the side of the channel, with poor lateral continuity and a short downstream extension distance. The horizontal distribution has certain random characteristics.

### Sand body architecture distribution pattern

**Figure 17:**
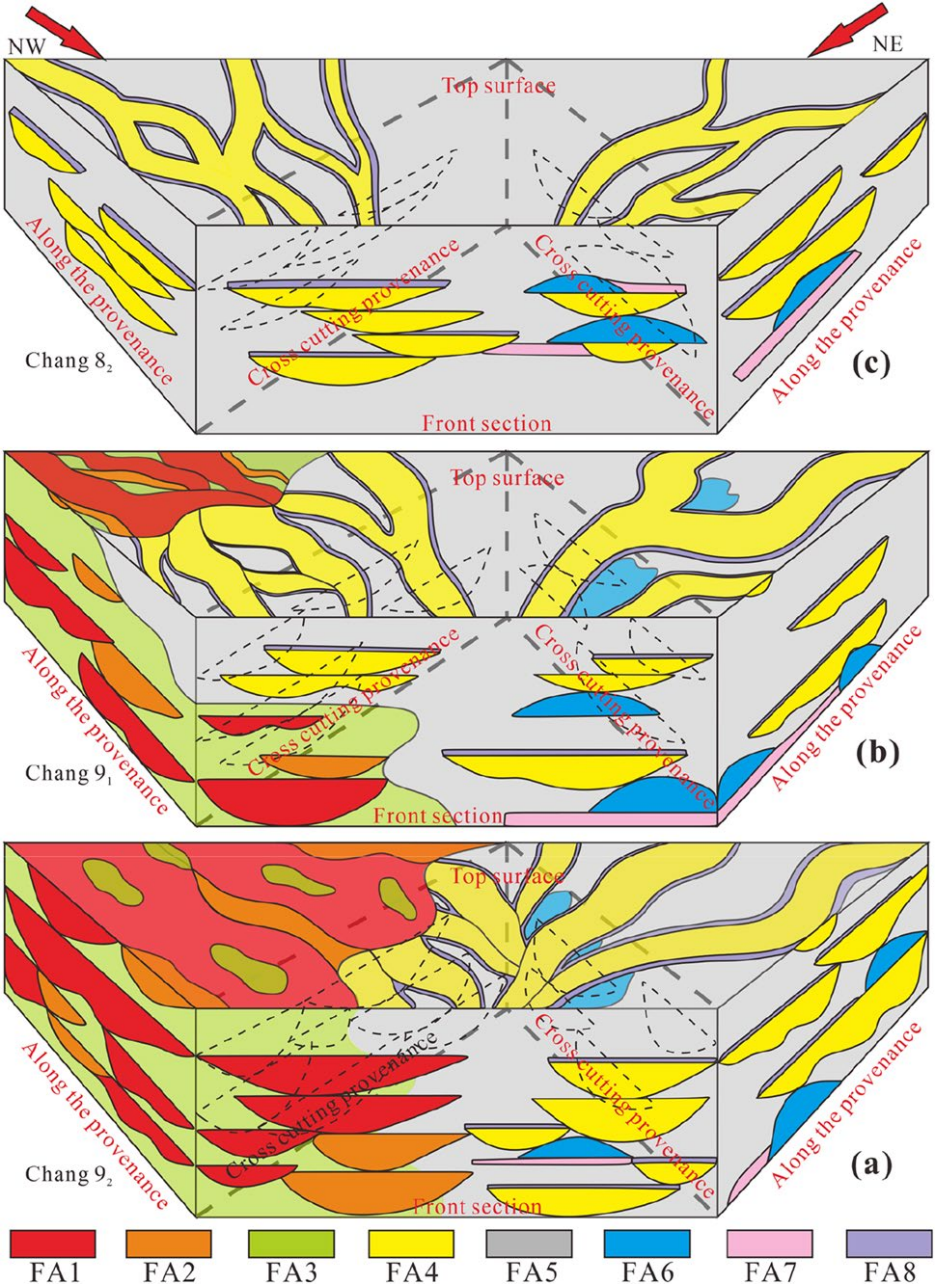
Spatial distribution pattern of all architectural elements.

Based on the above conclusions regarding architectural elements in the whole region and full consideration of the scale of the geometric forms of these elements and the spatial relationships between them, the spatial distribution mode of the architectural elements can be summarized through the ‘prism’ conceptual model (Fig. 17).

In the western lower sub-oil-bearing Chang 9_2_ formation, the sequence involves lateral splicing and vertical superposition of FA1 and FA2. FA3 is present between distributary channels as fine-grained sediments, and other sandy sediments occupy a large sedimentary volume (Fig. 17a). There are abundant lateral assemblages of architectural elements represented by black dotted lines in the direction perpendicular to the provenance direction, with positive cycle characteristics from bottom to top. In the provenance direction, FA4, FA5, and FA6 are the main architectural elements in the east. FA4 and FA6 are relatively developed and occupy a large sedimentary volume. FA5 is the argillaceous sediment of the distributary bay, and the architectural elements are closely superimposed. FA7 and FA8 are associated with each other. In the direction perpendicular to the provenance direction in the east, FA4 is mainly superimposed and spliced (black dotted line), and its abundance is significantly lower than that in the west. The front section reflects the characteristics of the east‒west intersection. Both sections are dominated by positive cycles, and they are gradually spliced from the bottom to the middle and are separated by FA1 and FA4 at the top. The top surface is the plane characterized by the deposition of FA1, FA2, and FA4. The western plain is extensive and intersects with the eastern front after forming the front.

The sub-oil-bearing Chang 9_1_ formation also continues the sedimentary pattern of Chang 9_2_, but the sedimentary environment of the western delta plain is mostly developed in the lower part and evolves to the delta front from the top (Fig. 17b). In general, deposits in the provenance and provenance-perpendicular directions are mainly composed of delta plain and delta front architectural elements, becoming a complete delta front suite in the east. In the provenance direction and provenance-perpendicular direction, the development of architectural elements reflects the trend of decreasing proportions of sandy sediments and increasing proportions of argillaceous sediments. The top surface reflects the bidirectional sedimentary evolution process. FA1 and FA2 in the west gradually change to FA4. The underwater distributary channel formed by FA4 in the east extends far, and FA6 is developed at the front estuary. There are few splices in the front section, and the continuity between the east and the west decreases.

The sedimentary environment of the shallow-water delta is present in the east and west of the sub-oil-bearing Chang 8_2_ formation (Fig. 17c). FA4 is mainly composed of sandy deposits. Due to the rise in lake level, FA5 occupies more volume, and the degree of superposition and splicing in the west is better than that in the east. FA6 is mostly developed in the east and is restricted in the west, and FA8 is mostly associated with FA4. FA7 is mainly visible in the east. The top surface shows that FA4 becomes thinner, and the hydrodynamic force decreases. There is less lateral splicing of architectural elements in the central eastern portion and western portion of the front section. The vertical superposition is mainly developed in the west, and the east presents the characteristics of the composite superposition of FA4 and FA6.

### Spatial distribution model of diagenetic facies under architectural constraints

Nine types of diagenetic facies were identified in the reservoirs in the study area. After mutual calibration of diagenetic facies and structural elements, it was found that different types of diagenetic facies have different positions within the skeletal elements. Therefore, the spatial distribution pattern of diagenetic facies can be explored from the perspective of differences in sedimentary genesis based on the constraints of architectural element analysis (Table 8).

**Table 8:** The main positions of different types of diagenetic facies in the architectural elements.

Type of diagenetic facies	FA1	FA2	FA4	FA6
Chang 8_2_–1 Chlorite lining residual intergranular pore diagenetic facies	–	–	FA4 bottom	FA6 middle and lower
Chang 8_2_–2 Illite-siliceous cement dissolution pore diagenetic facies	–	–	The upper part of FA4	–
Chang 8_2_–3 Chlorite filling and carbonate-cemented microporous diagenetic facies	–	–	–	FA6 lower part
Chang 8_2_–4 Carbonate-cemented compacted diagenetic facies	–	–	The upper part of FA4	–
Chang 9–1 Chlorite film and siliceous cement intergranular diagenetic facies	Central FA1	FA2 lower part	–	–
Chang 9–2 Chlorite film and lunate zeolite cement weakly dissolving diagenetic facies	–	–	The upper part of FA4	FA6 upper middle
Chang 9–3 Illite film intergranular diagenetic facies	–	–	The upper part of FA4	–
Chang 9–4 Lautonite cementation dissolution pore diagenetic facies	The upper part of FA1	The upper part of FA2	–	FA6 bottom
Chang 9–5 Carbonate-cemented dense diagenetic facies	–	–	FA4 bottom	FA6 bottom

**Figure 18:**
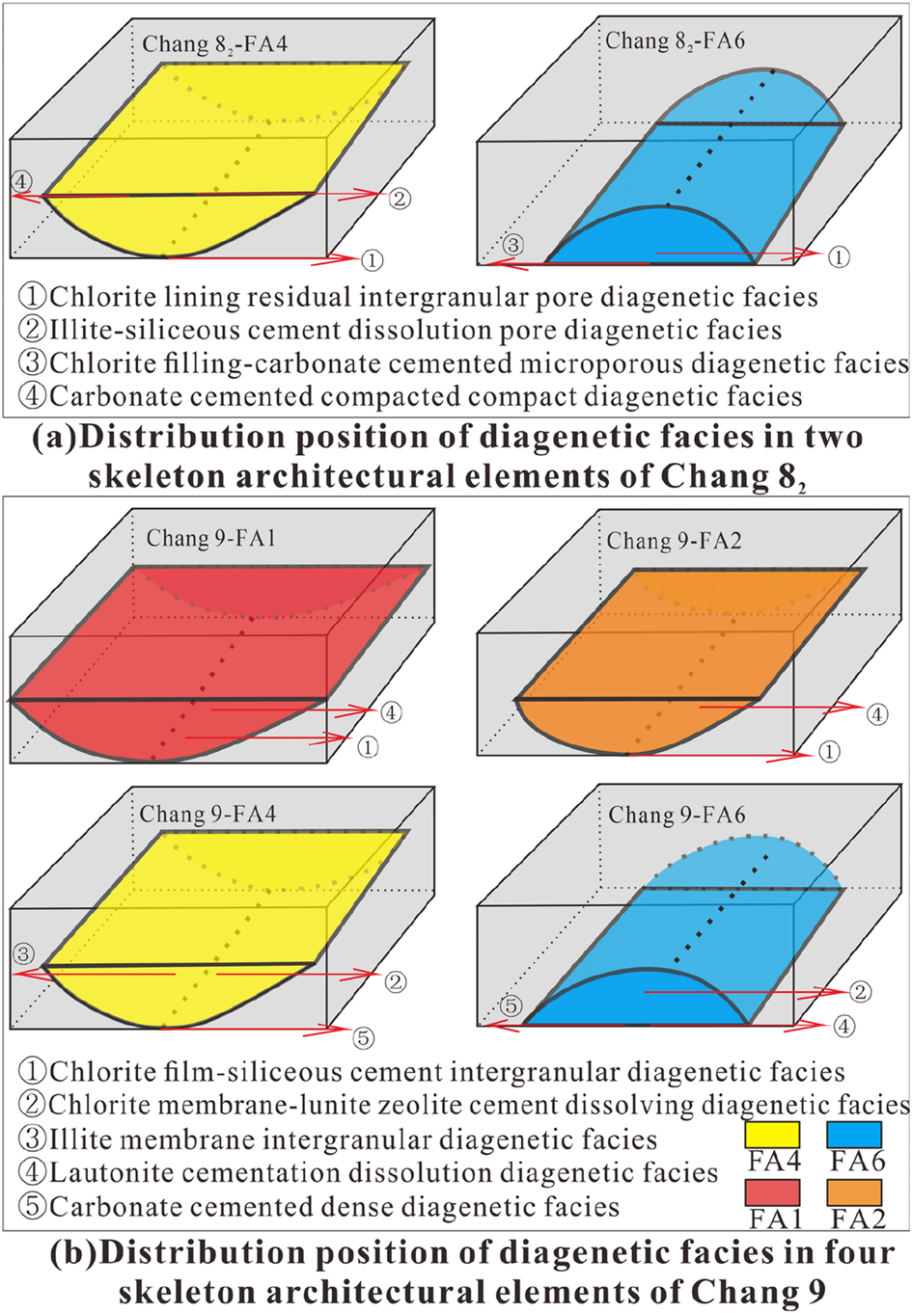
Distribution of diagenetic facies within the architectural elements.

Four types of diagenetic facies are distributed in the sub-oil-bearing Chang 8_2_ formation (Fig. 18a). Type 1 is a relatively high-quality diagenetic facies, which is usually developed at the bottom of FA4, reflecting large intergranular pores and good physical properties. The development in the lower part of FA6 is related to the sedimentary sequence of the estuarine bar. Because most estuarine bars are reverse cycle sedimentary sequences, the particle size increases upwards. However, due to the strong scouring and erosion present in shallow-water deltas, the upper part of the estuarine bar is easily eroded; therefore, only the lower part is preserved. Due to the development of illite and siliceous cementation and dissolution, type 2 develops in locations where more primary intergranular pores are preserved. For example, the upper part of FA4 is in relatively good contact with fluid, so it is a good development location.

Type 3 is a poor diagenetic facies. Due to the fine lithology at the bottom at the initial stage of the formation of the estuary bar, chlorite fills pores, and carbonate cementation leads to obvious pore reduction, resulting in the poor physical properties of the bottom of the estuary bars. Type 4 is a typical diagenetic facies of channel top sediments. The dissolution of fluid provides a material basis for calcareous cementation. Cementation occurs in an evaporative environment. After the arrival of a new stage of channel sediments, the compaction is enhanced, so the sediments remain well cemented and dense.

Five types of diagenetic facies are observed in the oil-bearing Chang 9 formation (Fig. 18b). For type 1, the development in the middle of FA1 is due to the large pore space from the lower part to the middle part of the braided channel configuration elements, a high number of intergranular pores are retained, and only a small amount of siliceous cementation occurs. The genesis of FA2 is similar to that of FA1, while the upper part usually features argillaceous material due to low-energy environments, and this material can be washed away and eroded. Therefore, the development position of this diagenetic facies is lower. The pore throat structure of the type 2 diagenetic facies is lower than that of the type 1 diagenetic facies and is mainly associated with intergranular corrosion pores, so this facies is mostly developed at the delta front, corresponding to FA4 and FA6. When an acidic fluid passes through, the laumontite cement is dissolved, so it is mostly present in upper positions with more contact with acidic fluids.

The type 3 diagenetic facies of is less common and can be seen only in the upper part of FA4. This is because the content of illite in Chang 9 is lower than that of chlorite, and the physical properties are still relatively good after the primary intergranular pores are preserved in a film shape. Therefore, this facies is related to the upper part of FA4. There are many types of the type 4 diagenetic facies, and examples can be seen in the upper parts of FA1 and FA2 and the bottom of FA6, which is related to the environment required for the dissolution of laumontite. Type 5 can be seen at the bottom of FA4 and FA6 because it is difficult to for acidic fluid dissolution to occur in these parts, and carbonate cement can be preserved.

**Figure 19:**
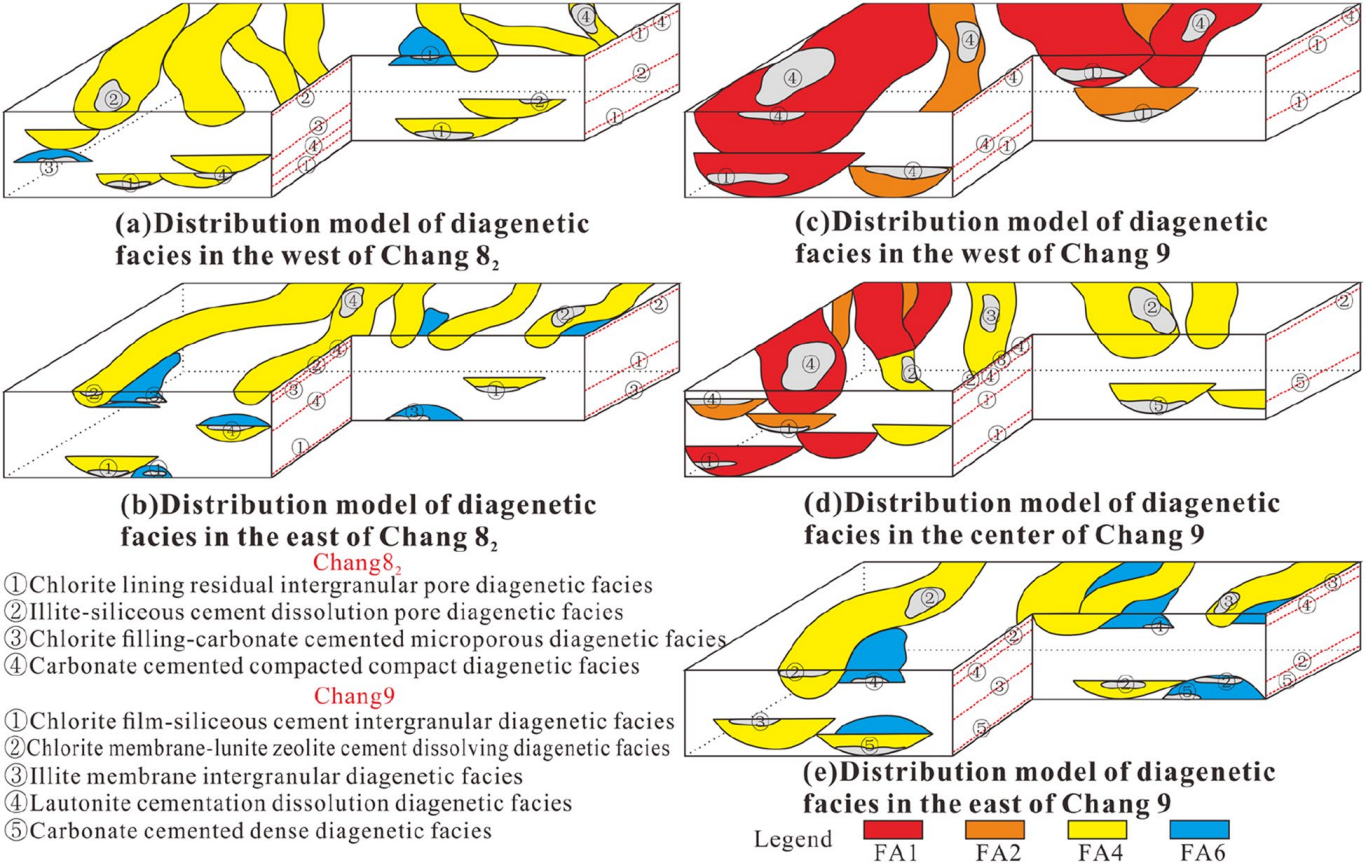
Distribution pattern of diagenetic facies constrained by architectural elements.

Because the spatial distribution of diagenetic facies is constrained by architectural elements, five typical areas are selected according to the sedimentary background of the study area, and the diagenetic facies model under architectural constraints is established. In this paper, two small regions in the west and east are selected for Chang 8_2_ (Fig. 19a, b), and three small regions in the west, middle, and east are selected for Chang 9 (Fig. 19c, d, e).

The shallow-water delta in the west of Chang 8_2_ is dominated by FA4, and FA6 is rare. Diagenetic facies type 4 is developed at the top, which is relatively unfavourable, while type 1 at the bottom of FA4 is more developed, so the lower part of this type is relatively favourable. Type 2 is observed in places, such as the top of FA4, which is related to siliceous development and dissolution. Type 3 is related to the development of estuarine bars, and the development at the bottom represents poor subsequent reservoir capacity (Fig. 19a).

The shallow-water delta in the east of Chang 8_2_ is also dominated by FA4, and FA6 is relatively common. In the vertical sequence, it is similar to the west, and FA4 and FA6 are superimposed. Therefore, types 1 and 3 can be located above and below each other, while type 2 is less common and can be seen only at the top of FA4, which is relatively favourable. The development of type 4 is the most unfavourable, and it can develop at the interface of architectural elements or at the top of an element and can seriously affect the continuity of a reservoir (Fig. 19b).

In the braided river delta plain in the west of Chang 9, the diagenetic facies are usually developed in the FA1 and FA2 architectural elements, and the top surface is mainly type 4. Therefore, the top surface of the architectural element combination exhibits unfavourable diagenetic facies. Type 1 is mostly distributed in the middle and lower parts of architectural elements; thus, the lower parts of braided channels and abandoned channels exhibit more favourable diagenetic facies than the upper part (Fig. 19c).

In the transition area from the braided river delta plain in the middle of Chang 9 to the front, the diagenetic facies are developed in various skeleton architectural elements. The distribution of the diagenetic facies at the top is mostly related to the dissolution of laumontite. When a chlorite film is developed, the diagenetic facies is relatively favourable (type 2). Types 1 and 5 are the most common in the vertical diagenetic facies arrangement. The diagenetic facies at the bottom of channel sedimentary architectural elements in a plain environment is relatively favourable, while the diagenetic facies at the bottom of FA4 in the front environment is unfavourable. The development of type 3 is low and can be seen at the top of FA4. Type 4 is mostly located in the upper part of the configuration elements, and there are few overlapping parts (Fig. 19d).

The front area of the meandering river delta in the east of Chang 9 shows that the diagenetic facies at the bottom of FA4 is relatively unfavourable (type 5), while in most of the vertical sequences, types 2, 3, and 4 alternate, with types 2 and 3 being more favourable for reservoir development. Type 4 can be seen in the lower part of FA6, which means that the lower part of the estuarine bar is vulnerable to laumontite cementation, which is unfavourable to reservoir development (Fig. 19e).

## Conclusion

1. After high-resolution sequence stratigraphic analysis, the sequence filling and dynamic response gradual process in the western part of the study area is A1 − C1 − C1 + C2, while that in the eastern part of the study area is C1 − C1 + C2 − C2 + A2.
2. Eight lithofacies types are identified-, and on this basis, eight FAs are identified, of which FA1, FA2, FA4, and FA6 are the skeletal architectural elements of the delta plain and front facies. In the vertical sequence of architectural elements, the delta plain is usually dominated by the vertical superposition of FA1 and FA2, while the delta front exhibits two modes: the superposition of FA4 and the combined superposition of FA4 and FA6.
3. In the lateral combination relationship of architectural elements, the delta plain is dominated by FA1 and FA2 lateral cutting, and FA1 and FA4 lateral cutting can be seen locally. The delta front mainly presents the combination relationship of FA4 mutual lateral cutting and FA4 and FA6 lateral cutting. From bottom to top, the degree of superposition and combination of eight skeletal architectural elements worsens progressively, and the development scale and degree of these elements in the western braided river delta system are better than those in the eastern meandering river delta system.
4. In the architectural distribution model, affected by different sedimentary areas, these elements show significant variability in their spatial combination. From bottom to top, there are the following progressive relationships: strong FA1 + FA2 combination relationship → weak FA1 + FA2 combination relationship with FA4 → strong FA4 individual combination relationship. From west to east, the hydrodynamic strength tends to weaken slowly; thus, FA1 + FA2 → FA1 + FA2 + FA4 → FA4 → FA4 + FA6.
5. After matching the distribution of diagenetic facies with the development of architectural elements, this study found that the types of diagenetic facies dominated by intergranular pores and dissolution pores are mostly located at the bottom or in the lower to middle parts of the skeletal elements. Relevant measures for tapping the potential of remaining oil can be formulated based on the combination of architectural element types and the distribution of diagenetic facies.

## Data Availability

The authors declare that all the data in the submitted manuscript are freely available to any researcher wishing to use them for non-commercial purposes, without breaching participant confidentiality. Please contact the corresponding author directly if someone requests the data from this study.
